# The Progeny of *Arabidopsis thaliana* Plants Exposed to Salt Exhibit Changes in DNA Methylation, Histone Modifications and Gene Expression

**DOI:** 10.1371/journal.pone.0030515

**Published:** 2012-01-23

**Authors:** Andriy Bilichak, Yaroslav Ilnystkyy, Jens Hollunder, Igor Kovalchuk

**Affiliations:** 1 Department of Biological Sciences, University of Lethbridge, Lethbridge, Alberta, Canada; 2 Department of Plant Systems Biology, Vlaams Instituut voor Biotechnologie, Ghent University, Ghent, Belgium; 3 Department of Molecular Genetics, Ghent University, Ghent, Belgium; Temasek Life Sciences Laboratory, Singapore

## Abstract

Plants are able to acclimate to new growth conditions on a relatively short time-scale. Recently, we showed that the progeny of plants exposed to various abiotic stresses exhibited changes in genome stability, methylation patterns and stress tolerance. Here, we performed a more detailed analysis of methylation patterns in the progeny of *Arabidopsis thaliana* (Arabidopsis) plants exposed to 25 and 75 mM sodium chloride. We found that the majority of gene promoters exhibiting changes in methylation were hypermethylated, and this group was overrepresented by regulators of the chromatin structure. The analysis of DNA methylation at gene bodies showed that hypermethylation in the progeny of stressed plants was primarily due to changes in the 5′ and 3′ ends as well as in exons rather than introns. All but one hypermethylated gene tested had lower gene expression. The analysis of histone modifications in the promoters and coding sequences showed that hypermethylation and lower gene expression correlated with the enrichment of H3K9me2 and depletion of H3K9ac histones. Thus, our work demonstrated a high degree of correlation between changes in DNA methylation, histone modifications and gene expression in the progeny of salt-stressed plants.

## Introduction

Living organisms are frequently influenced by abiotic and biotic environmental factors. Apart from physiological changes in the exposed generation, stress also alters epigenetic marks that can potentially persist in the progeny. Epigenetic factors can contribute to both short-term (mitotic) and long-term (meiotic) inheritance of an altered gene expression without changing the primary DNA sequences [1]. The key factors that are implicated in epigenetic memory include, but are not limited to, DNA cytosine methylation, post-translational histone modifications and metabolism of small RNA molecules that can interact to form self-reinforcing loops [2], [3], [4]. DNA methylation is largely responsible for regulating the transcriptional genome output as well as for directing the deposition of other epigenetic marks and chromatin remodelling [5]. Overall, slightly more than 20% of the Arabidopsis genome is methylated, with transposable elements (TEs) and DNA repeats representing the largest fraction of methylated sequences. Whereas TEs are heavily methylated throughout their whole sequence, non-TE genes that are expressed in a tissue-specific manner are primarily methylated at the gene promoter regions [6]. At the same time, methylation of coding regions does not usually result in gene silencing [6], [7]. Methylation of transcribed regions seems to primarily occur at CG sites, and there appears to be no obvious correlation between the level of gene-body methylation and gene expression [5]. Genes methylated within the coding sequence display moderate expression levels and are less likely to have tissue-specific expression [5], [6], [8]. Methylation in the coding sequence of these genes moderately correlates with the level of gene expression [9].

Alterations in DNA methylation have been suggested to be involved in the process of adaptation to stress in plants [10], [11], [12], [13]. Our previous research also showed that stress exposure resulted in changes in DNA methylation and gene expression in unexposed progeny [14], [15]. The persistence of cytosine methylation and its reversibility makes it an ideal mechanism controlling transgenerational response to stress. DNA methylation was also shown to direct the deposition of certain chromatin marks such as differentially modified histones. The analysis of the DNA methyltransferase and histone methyltransferase mutants revealed a tight link between DNA methylation and post-translational histone modifications [16], [17], [18], suggesting that epigenetic regulation of gene expression is a complex mechanism of interaction between chromatin remodelling factors. In a mutant of DECREASE IN DNA METHYLATION1 (DDM1) which is responsible for the maintenance of cytosine DNA methylation in the heterochromatic regions, a decrease in DNA methylation is associated with gain of H3K4me and loss of H3K9me [19]. Additionally, the copia-like elements (TA2 and TA3) lose the H3K9me modification in the CHROMOMETHYLASE3 (CMT3) and DNA METHYLTRANSFERASE (MET1) double mutants [20]. On the contrary, mutations of the KRYPTONITE gene that encodes a member of the Su(var)3-9 family of histone methyltransferases causes depletion of H3K9me, loss of DNA methylation, and lower gene silencing [21].

Histone modifications provide another layer of epigenetic information that responds to the developmental and environmental cues in a fast and efficient manner. Among various histone modifications, histone acetylation acts directly by loosening histone association with DNA leading to transcriptional activation, whereas histone methylation helps recruit other effector proteins and their complexes, and thus either activating or repressing gene expression. For example, modifications at H3K9 have positive and negative effects on gene expression; whereas acetylation at H3K9 correlates with high gene expression and dimethylation of H3K9 acts as a repressive chromatin mark [9].

A correlation between gene expression, DNA methylation and histone modifications is not always obvious. Zhou et al. (2010) analyzed the genome-wide distribution of acetylation and demethylation of histone H3 lysine 9 (H3K9ac and H3K9me2) and correlated it with gene expression data [22]. They found that high levels of H3K9ac were primarily associated with actively transcribed genes and infrequently associated with transposons. In contrast, H3K9me2 was found to be primarily targeting TEs and occasionally – poorly transcribed non-TE genes. The authors found H3K9ac to cluster around transcription and translation start sites, whereas H3K9me2 was shown to span the entire coding region [22]. Lang-Mladek et al. (2010) analyzed changes in DNA methylation, histone acetylation and gene expression in response of somatic Arabidopsis tissue to heat stress [23]. The authors found a positive correlation between changes in the level of gene expression and histone acetylation at a given locus but did not observe any correlation between the levels of gene expression and methylation. Unfortunately to date, no analysis of histone modifications in the progeny of stressed plants has been performed. Thus, there is no evidence whether changes in DNA methylation in progenies of stressed plants correlate with changes in histone post-translational modifications.

Here, we extended our previous work by performing a more detailed analysis of DNA methylation of progenies of salt-stressed plants. We followed this work by the analysis of histone modifications at a set of selected promoter- and gene-coding regions. Furthermore, we analyzed the expression of these genes and performed a correlation analysis of methylation patterns, gene expression and histone modifications. We found a high degree of correlation among the levels of methylation, histone modification status, and the level of mRNA in *SUVH2*, *SUVH5*, *SUVH6*, *SUVH8*, *UBP26*, *DRB2*, *WRKY22*, *ROS1*, *MSH6*, *UVH3* homolog, *APUM3* and *MOS6* in the progeny of salt-stressed plants. Our findings support previous reports on transgenerational changes in plants [24], [25], they also provide new evidence of a tight correlation between epigenetic marks involved in stress response.

## Results

### The Progeny of Stressed Plants Exhibit Changes in DNA Methylation

Our previous methylation analysis using cytosine extension assay showed that the genome of the progeny of stressed plants was hypermethylated in “25 mM plants” and “75 mM plants” by 12% and 10%, respectively.

**Figure 1:**
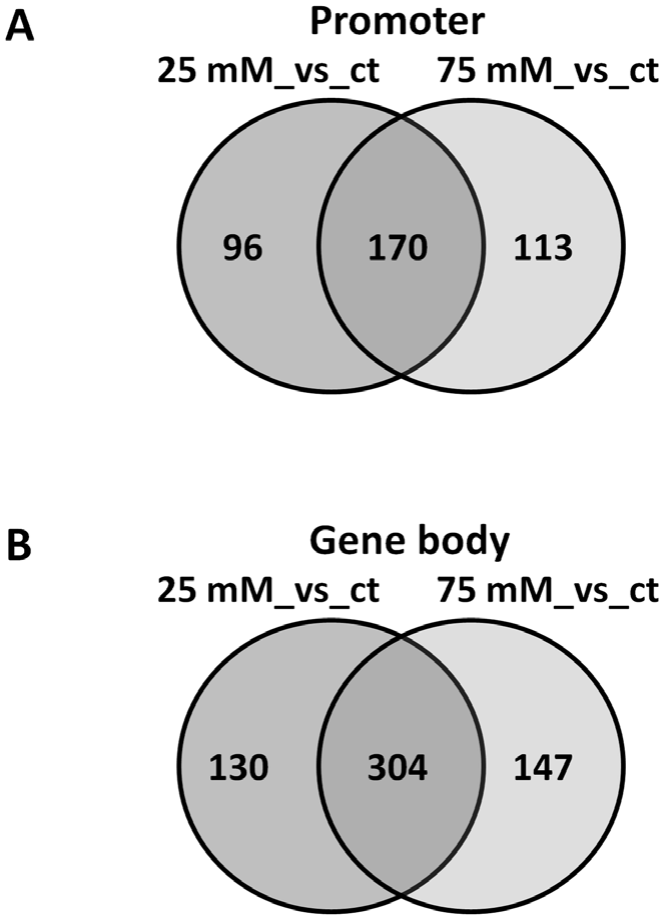
Venn diagrams showing the number of the similarly and differentially methylated promoter regions (A) and gene body regions (B) in the progeny of plants exposed to 25 and 75 mM NaCl as compared to the progeny of control plants.

To gain more detailed knowledge about a type of sequences in which changes in DNA methylation occurred, we analyzed methylation at the promoter and transcribed regions of all genes located on the NimbleGen Array #2. First, for the analysis of the promoter region, we used the 5 kb sequence 5′ of a transcribed region. For the analysis of methylation at the transcribed region, we used the entire sequences of the transcribed region of each gene. We identified the number of methylated reporters (the region of 90 nt in length, see Materials and Methods for details) out of the total number of reporters which are present either in the 5-kb promoter region or in the transcribed region and compared these data between the progeny of control and stressed plants. To obtain a list of differentially methylated promoters and gene-body regions, we considered the regions to be hypermethylated if methylation changed from 0–50% in the progeny of control plants to 50–100% in the progeny of stressed plants. Similarly, we considered the regions to be hypomethylated if methylation changed from 50–100% in the progeny of control plants to 0–50% in the progeny of stressed plants. Out of 6,763 promoters and transcribed regions analyzed, there were 266 and 283 promoter regions in which methylation changes were observed in the progeny of plants exposed to 25 and 75 mM NaCl, respectively, as compared to the progeny of control plants; 170 promoter regions were similarly regulated in both plant groups exposed to 25 and 75 mM NaCl (Figure 1A). There were 434 and 451 differentially methylated gene-body regions in 25 and 75 mM plant groups, respectively; 304 regions were similar for both groups (Figure 1B). A comprehensive list of all hypermethylated and hypomethylated promoters and genes is shown on Figures S1, S2.

To analyze whether differences in methylation between the progeny of stressed and control plants were significant, we performed the non-parametric statistical Wilcoxon rank-sum test using ranked data for ct, 25 mM and 75 mM plants groups in three regions: the promoter regions, the gene body regions and all regions. The analysis of a 1 percent tail in hypomethylated regions (the start of the rank) showed that neither 25 mM nor 75 mM plant groups were different from ct plants, although they were different from each other (Table S1). The analysis of a 1 percent tail in hypermethylated regions (the end of the rank) showed that both the 25 mM and 75 mM plant groups were different from ct plants, and the 25 mM and 75 mM plant groups were mostly similar to each other (Table S1). Further analysis showed that these similarities in hypomethylated regions and significant differences in hypermethylated regions were preserved even for a 10 percent tail (Table S2).

**Figure 2:**
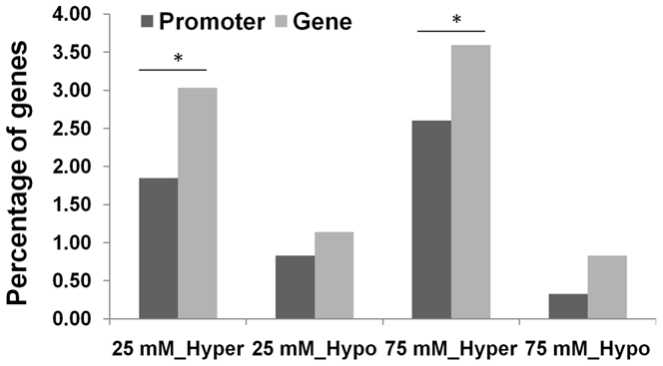
Percentage of differentially methylated genes. The figure shows the percentage of genes that are hyper- or hypomethylated at the promoter or gene body regions in the progeny of stressed (25 or 75 mM) plants as compared to the progeny of non-stressed control plants. “25_Hypo” and “75_Hypo” – stand for hypomethylated regions in the progeny of plants exposed to 25 and 75 mM NaCl, respectively. “25_Hyper” and “75_Hyper” – represent hypermethylated regions in the progeny of plants exposed to 25 and 75 mM NaCl, respectively. The asterisks denote a significant difference between the percentage of hypermethylated and hypomethylated regions (p<0.05; single-factor Anova).

While comparing the lists of hyper- and hypomethylated regions, we found that the majority of genes and promoters exhibiting methylation changes in the progeny of stressed plants were hypermethylated. Namely, there was a 2.5-fold higher percentage of hypermethylated genes compared to hypomethylated genes in the progeny of plants exposed to 25 mM NaCl (p = 0.045) and a 5-fold higher percentage – in the progeny of plants exposed to 75 mM NaCl (p = 0.003) (Figure 2A).

To further decrease the number of genes which have differentially methylated promoters, we decided to restrict the promoter size to a 1,000-nt sequence upstream of the transcribed region. A comparison of methylation levels between the progeny of stressed and control plants showed 18 hypermethylated and 6 hypomethylated promoters exhibiting over 50% changes in methylation (Table 1, Table 2). Methylation changes were similar in the progeny of plants exposed to 25 and 75 mM NaCl in 12 out of 24 promoters being analyzed (Table 1, Table 2).

**Table 1:** Promoters hypermethylated in the progeny of stressed plants.

AGI	N of reporters	N of methylated reporters			% of methylated reporters			Gene symbol(function)
		ct	25	75	ct	25	75	
AT2G36490	11	0	6	0	0	55	0	ROS1
AT2G47275	7	0	0	5	0	0	71	MICRORNA403 (MIR403)
AT2G25930	10	0	0	7	0	0	70	EARLY FLOWERING 3 (ELF3)
AT2G24740	9	0	0	6	0	0	67	SU(VAR)3-9 HOMOLOG 8 (SUVH8)
AT2G35160	7	0	0	5	0	0	71	SU(VAR)3-9 HOMOLOG 5 (SUVH5)
AT2G45650	10	0	0	9	0	0	90	AGAMOUS-LIKE 6 (AGL6)
AT3G55970	2	0	0	2	0	0	100	JASMONATE-REGULATED GENE 21 (JRG21)
AT3G49430	10	0	8	6	0	80	60	Ser/Arg-rich protein 34a (SRp34a)
AT3G50500	10	0	5	6	0	50	60	SNF1-RELATED PROTEIN KINASE 2.2 (SNRK2.2)
AT3G48057	12	0	11	11	0	92	92	MICRORNA843A (MIR843A)
AT3G25770	9	0	5	4	0	56	44	ALLENE OXIDE CYCLASE 2 (AOC2)
AT3G20340	9	0	9	8	0	100	89	downregulated by paraquat
AT3G23100	8	0	0	6	0	0	75	XRCC4
AT3G63010	7	0	0	5	0	0	71	GA INSENSITIVE DWARF1B (GID1B)
AT4G02150	10	0	8	6	0	80	60	MODIFIER OF SNC1, 6 (MOS6)
AT4G02070	10	0	7	6	0	70	60	MUTS HOMOLOG 6 (MSH6)
AT4G04695	10	0	0	8	0	0	80	(CPK31)
AT4G01250	10	0	0	5	0	0	50	(WRKY22)

The table shows the list of the genes that were hypermethylated at the promoter region. The promoter regions were defined as 1,000 nucleotides. The total number of reporters shows the number of reporters located on the array. The number of methylated reporters is the number of reporters for which the difference between enriched and input DNA was observed (see Materials and Methods for details). The percentage of methylated reporters reflects the percentage of reporters in which methylation has changed.

**Table 2:** Promoters hypomethylated in the progeny of stressed plants.

AGI	N of reporters	N of methylated reporters			% of methylated reporters			Gene symbol(function)
		ct	25	75	ct	25	75	
AT2G25820	11	6	0	0	55	0	0	DREB subfamily A-4 of ERF/AP2 transcription factor family
AT2G28550	10	8	0	0	80	0	0	RELATED TO AP2.7 (RAP2.7)
AT3G46710	7	4	0	0	57	0	0	disease resistance protein (CC-NBS-LRR class)
AT3G48900	11	7	0	0	64	0	0	DNA repair/chromatin binding (UVH3 homolog)
AT3G23240	11	7	0	0	64	0	0	ETHYLENE RESPONSE FACTOR 1 (ERF1)
AT3G61650	10	6	0	2	60	0	20	GAMMA-TUBULIN (TUBG1)

The table shows the list of the genes that were hypomethylated at the promoter region. The promoter regions were defined as 1,000 nucleotides. The total number of reporters shows the number of reporters located on the array. The number of methylated reporters is the number of reporters for which the difference between enriched and input DNA was observed (see Materials and Methods for details). The percentage of methylated reporters reflects the percentage of reporters in which methylation has changed.

Similarly, to restrict the number of genes which were differentially methylated at the gene body, we considered only those genes with an over 80% increase or decrease in methylation, that is from 0–20% in the progeny of control to 80–100% of methylation in the progeny of stressed or from 80–100% in the progeny of control to 0–20% of methylation in the progeny of stressed. The analysis showed that there were 15 and 7 genes hypermethylated and hypomethylated at the transcribed regions, respectively (Table 3, Table 4). This again indicated that hypermethylation prevailed in the progeny. In 14 out of 22 genes being analyzed, hypermethylations or hypomethylations of a given gene were observed in the progeny of plants exposed to both 25 and 75 mM NaCl (Table 3, Table 4).

**Table 3:** Genes hypermethylated in the progeny of stressed plants.

AGI	N of reporters	N of methylated reporters			% of methylated reporters			Gene symbol(function)
		ct	25	75	ct	25	75	
AT2G28380	27	0	15	12	0	56	44	DSRNA-BINDING PROTEIN 2 (DRB2)
AT2G29140	45	0	23	16	0	51	36	Arabidopsis Pumilio 3 (APUM3)
AT2G42080	25	0	15	14	0	60	56	DNAJ heat shock protein
AT2G23740	65	0	57	56	0	88	86	SU(VAR)3-9 HOMOLOG 6 (SUVH6)
AT2G33290	28	0	0	18	0	0	64	SU(VAR)3-9 HOMOLOG 2 (SUVH2)
AT2G33340	55	0	33	31	0	60	56	MOS4-ASSOCIATED COMPLEX 3B (MAC3B)
AT2G23380	53	0	0	27	0	0	51	CURLY LEAF (CLF)
AT3G48050	63	0	39	35	0	62	56	DNA binding; Transcription elongation factor
AT3G11450	25	0	19	15	0	76	60	DNAJ heat shock protein, MYB-like
AT3G44880	31	0	18	17	0	58	55	ACCELERATED CELL DEATH 1 (ACD1)
AT3G49600	70	0	37	32	0	53	46	UBIQUITIN-SPECIFIC PROTEASE 26 (UBP26)
AT3G03420	19	0	11	10	0	58	53	Ku70-binding family protein
AT4G00450	83	0	66	65	0	80	78	CRYPTIC PRECOCIOUS (CRP)
AT4G04340	46	0	34	32	0	74	70	early-responsive to dehydration protein-related
AT4G08210	24	0	23	22	0	96	92	pentatricopeptide (PPR) repeat-containing protein

The table shows the list of the genes that were hypermethylated at the gene body region. The total number of reporters shows the number of reporters located on the array. The number of methylated reporters is the number of reporters for which the difference between enriched and input DNA was observed (see Materials and Methods for details). The percentage of methylated reporters reflects the percentage of reporters in which methylation has changed.

**Table 4:** Genes hypomethylated in the progeny of stressed plants.

AGI	N of reporters	N of methylated reporters			% of methylated reporters			Gene symbol(function)
		ct	25	75	ct	25	75	
AT3G32316	10	5	3	0	50	30	0	AGAMOUS homolog
AT3G28925	20	10	0	8	50	0	40	ATSMC3 (ARABIDOPSIS THALIANA STRUCTURAL MAINTENANCE OF CHROMOSOME 3)
AT3G07520	38	19	0	0	50	0	0	GLUTAMATE RECEPTOR 1.4 (GLR1.4)
AT3G50360	15	8	7	0	53	47	0	CENTRIN2 (ATCEN2)
AT3G15790	17	10	9	0	59	53	0	METHYL-CPG-BINDING DOMAIN 11 (MBD11)
AT4G04920	74	48	0	34	65	0	46	SENSITIVE TO FREEZING 6 (SFR6)
AT4G02460	50	29	0	28	58	0	56	POSTMEIOTIC SEGREGATION 1 (PMS1)

The table shows the list of the genes that were hypomethylated at the gene body region. The total number of reporters shows the number of reporters located on the array. The number of methylated reporters is the number of reporters for which the difference between enriched and input DNA was observed (see Materials and Methods for details). The percentage of methylated reporters reflects the percentage of reporters in which methylation has changed.

Since the cultivar used in this study was C24 and NimbleGen array was based on the sequence of cultivar Columbia, there was a possibility that substantial polymorphism would interfere with hybridization. We analyzed sequence polymorphism for 8 shortlisted genes and found an average of 1.6 substitutions in the average sequence length of ∼3,500 nt (Table S3). Such a low percentage (0.05%) of sequence polymorphism unlikely interfered with hybridization between C24 DNA and Columbia DNA-based NimbleGen array.

### An Increase in Methylation in the Progeny of Stressed Plants is Primarily due to Changes in the Exons and at the 5′ or 3′ Ends of the Genes

**Figure 3:**
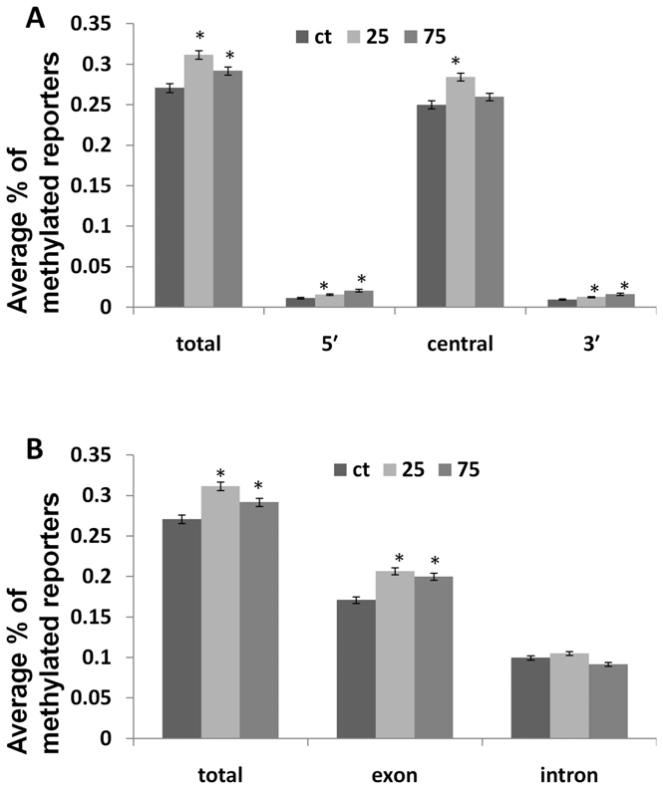
The average percentage of the differentially methylated reporters located in various parts of the gene body. The figure shows the average percentage of methylated reporters (with SE, calculated for over 2,000 genes) found in the coding sequences of genes in the progeny of control plants (Ct) and the progeny of stressed plants (25 and 75 mM). The asterisks denote a significant difference between the progeny of plants exposed to 25 or 75 mM NaCl and the progeny of control plants (p<0.05). A. The average percentage of methylated reporters in the entire coding sequence, in the 300 nts of the 5′ end, in the 300 nts of the 3′ end, and in the central part of the gene. B. The average percentage of methylated reporters in the entire coding sequence, in the exon and intron regions of the gene.

Next, we tested whether there is a difference between the methylation level of the 5′ end, the central part and the 3′ end of the gene. Previous reports suggested that methylation in the coding regions differs, with central parts of the gene typically having higher methylation levels [9]. The genes that are methylated in the central part of the gene body are typically moderately expressed, and methylation levels correlate positively with gene expression [9]. Our analysis of methylation of 2,317 genes showed that the 5′ and 3′ ends of the genes (300-nt from either side) had approximately 25-fold lower levels of methylation as compared to the central part of the gene analyzed in control plants (Figure 3A; Table 5). We also found that the differences in methylation levels of the progeny of stressed plants as compared to the progeny of control plants were much more dramatic in the 5′ and 3′ ends of the genes rather than in the central part (Figure 3A, Table 5). Methylation levels in the entire gene body were 15.1% and 7.8% higher in the progeny of plants exposed to 25 and 75 mM NaCl, respectively, as compared to control plants. Methylation levels in the central part of the genes were 13.7% and 3.9% higher in the progeny of stressed plants exposed to 25 and 75 mM NaCl. On the contrary, in the 300-nt region of the 5′ end of the gene, methylation levels was 37.3% and 81.5% higher in plants exposed to 25 and 75 mM NaCl, whereas at the 3′ end, they were 37.6% and 69% higher.

**Table 5:** Statistical analysis of differences in methylation levels in different parts of the gene body.

*Source of variation*		*Fold difference*	*F*	*P-value*	*F crit*
TOTAL	25mM_ct	1.15	30.47	3.58E-08	3.84
	75mM_ct	1.08	8.30	0.004	3.84
	75mM_25mM	0.94	7.64	0.006	3.84
5′ end	25mM_ct	1.37	9.97	0.002	3.84
	75mM_ct	1.82	22.79	1.87E-06	3.84
	75mM_25mM	1.32	6.04	0.014	3.84
Middle	25mM_ct	1.14	24.89	6.28E-07	3.84
	75mM_ct	1.04	2.16	0.140	3.84
	75mM_25mM	0.91	13.84	2.00E-04	3.84
3′ end	25mM_ct	1.32	6.59	0.010	3.84
	75mM_ct	1.69	14.31	1.57E-04	3.84
	75mM_25mM	1.28	3.75	0.053	3.84
Exon	25mM_ct	1.21	34.12	5.54E-09	3.84
	75mM_ct	1.17	13.16	2.88E-04	3.84
	75mM_25mM	0.97	5.22	0.0224	3.84
*Intron*	25mM_ct	1.05	7.68	0.006	3.84
	75mM_ct	0.92	1.28	0.258	3.84
	75mM_25mM	0.84	16.07	6.2E-05	3.84

Single-factor ANOVA was used to identify significant differences between the progeny of plants exposed to 25 mM NaCl and the progeny of control plants (25mM_ct), between the progeny of plants exposed to 75 mM NaCl and the progeny of control plants (75mM_ct), and between the progeny of plants exposed to 75 mM NaCl and the progeny of plants exposed to 25 mM NACl (75mM_25mM). The analysis was performed for the entire sequence of the gene body as well as for the 5′/3′ ends, the middle part, exon/intron regions.

Recent data on the analysis of DNA methylation in exons versus introns showed that methylation levels in exons are generally higher, and this may contribute to either exon definition or control of alternative gene splicing [26], [27]. We analyzed whether methylation in the exon or intron of the genes differs in the progeny of stressed and control plants. We found that, in general, methylation levels in the exons were over 70% higher than those in the introns. A comparison between progenies of stressed and non-stressed plants showed that the increase in methylation in the progeny of salt-stressed plants was mainly due to changes in the exons. In the exons of the progeny of plants exposed to 25 and 75 mM NaCl, the level of hypermethylation was 21% and 17%, respectively. In the introns of plants exposed to 25 mM NaCl the level of hypermethylation was 5%, whereas in the introns of plants exposed to 75 mM NaCl we observed 8% hypomethylation, as compared to those in control plants (Figure 3B; Table 5).

### The Progeny of Stressed Plants Have a Large Number of Hypermethylated Genes Involved in the Regulation of Chromatin Structure

While analyzing the aforementioned list of genes, we noticed that a great number of them were involved in the regulation of chromatin structure. For example, genes that encode histone methyltransferases (HMTases), namely, *SUVH2*, *SUVH5*, *SUVH8*, were highly hypermethylated in the promoter region, the transcribed region or both in the progeny of exposed plants. SUVH2 is one of the main players among HMTases; together with SUVH4, it significantly contributes to mono- and dimethylation of H3K9 [28] and heterochromatic gene silencing [17]. The SUVH5 protein has the weak HMTase activity and is involved in methylation of H3K9 and CHROMOMETHYLTRANSFERASE3 (CMT3) - mediated non-CG methylation *in vivo*. A similar trend of hypermethylation in the coding region in the progeny of salt-stressed plants was observed in the *UBP26* gene. UBP26 and SUP32 catalyze H2B deubiquitination, and UBP26 is also required for heterochromatic histone H3 methylation and DNA methylation [29]. The gene encoding a Polycomb repressive complex 2 (PRC2) subunit, CURLY LEAF (CLF), a histone-lysine N-methyltransferase, was also hypermethylated. A decrease in the CLF activity results in early flowering [30].Intriguingly, genes that are involved in the transcriptional and posttranscriptional regulation of gene expression are also affected by stress conditions. For example, *ROS1*, a repressor of transcriptional gene silencing, also showed high levels of hypermethylation in the promoter region in “25 mM” and “75 mM” plants. The *ROS1* gene encodes a DNA glycosylase that functions by demethylating the target promoter DNA and, as a result, protects genes from potentially deleterious methylation [31]. Additionally, high levels of hypermethylation in the coding regions were observed in genes that are involved in post-transcriptional regulatory events – DOUBLE STRANDED RNA - BINDING PROTEIN (DRB2) and ARABIDOPSIS PUMILIO (APUM3). Arabidopsis DRB2, possibly, cooperates with DCL1 in specific tissues to mediate the metabolism of a subset of miRNAs [32]. APUM3 belongs to the Puf family proteins that have important roles in controlling gene expression at the post-transcriptional level by promoting RNA decay and repressing translation. The Pumilio homology domain (PUM-HD) is a conserved region within Puf proteins that binds with sequence specificity to the 3′ untranslated region (UTR) of target mRNAs. It was suggested that these proteins might be involved in a wide range of post-transcriptional regulatory events allowing plants to respond rapidly to changes of environmental conditions [33].

To confirm the methylation data obtained by MeDIP, we performed MeDIP-qPCR analysis for promoter and gene body regions of *SUVH5*, *SUVH6*, *MSH6*, *WRKY22*, *UBP26* and *UVH3* homolog. We partially confirmed methylation changes in all of these genes (Figure S3).We noted that MeDIP-qPCR and MeDIP data were more similar for the progeny of 25 mM stress as compared to 75 mM.

### Changes in DNA Methylation Correlate with Changes in Histone Modifications

**Figure 4:**
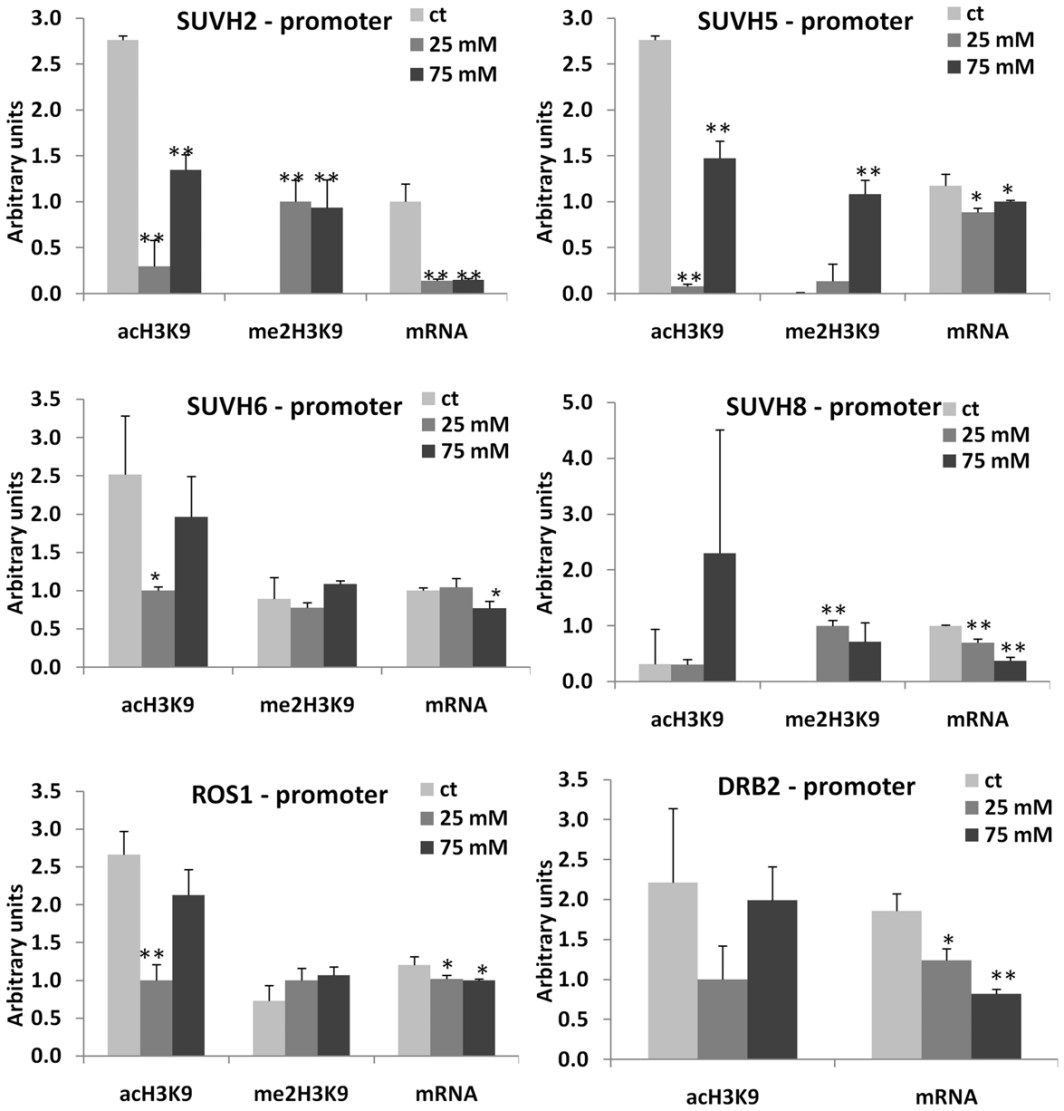
Histone modifications (H3K9me2 and H3K9ac) at the promoter regions of *SUVH2*, *SUVH5*, *SUVH6*, *SUVH8*, *ROS1* and *DRB2* genes. The figure shows the levels of H3K9me2 and H3K9ac observed at the promoter region of *SUVH2*, *SUVH5*, *SUVH6*, *SUVH8*, *ROS1* and *DRB2* genes. Each figure also shows mRNA levels for each of the genes. The Y-axis shows the levels of mRNA expression and H3K9me2/H3K9ac in average arbitrary units (calculated from three independent experiments with SD). The asterisks denote a significant difference between the progeny of stressed (25 and 75 mM) and control plants; one asterisk stands for p<0.05 and two asterisks for p<0.01.

**Figure 5:**
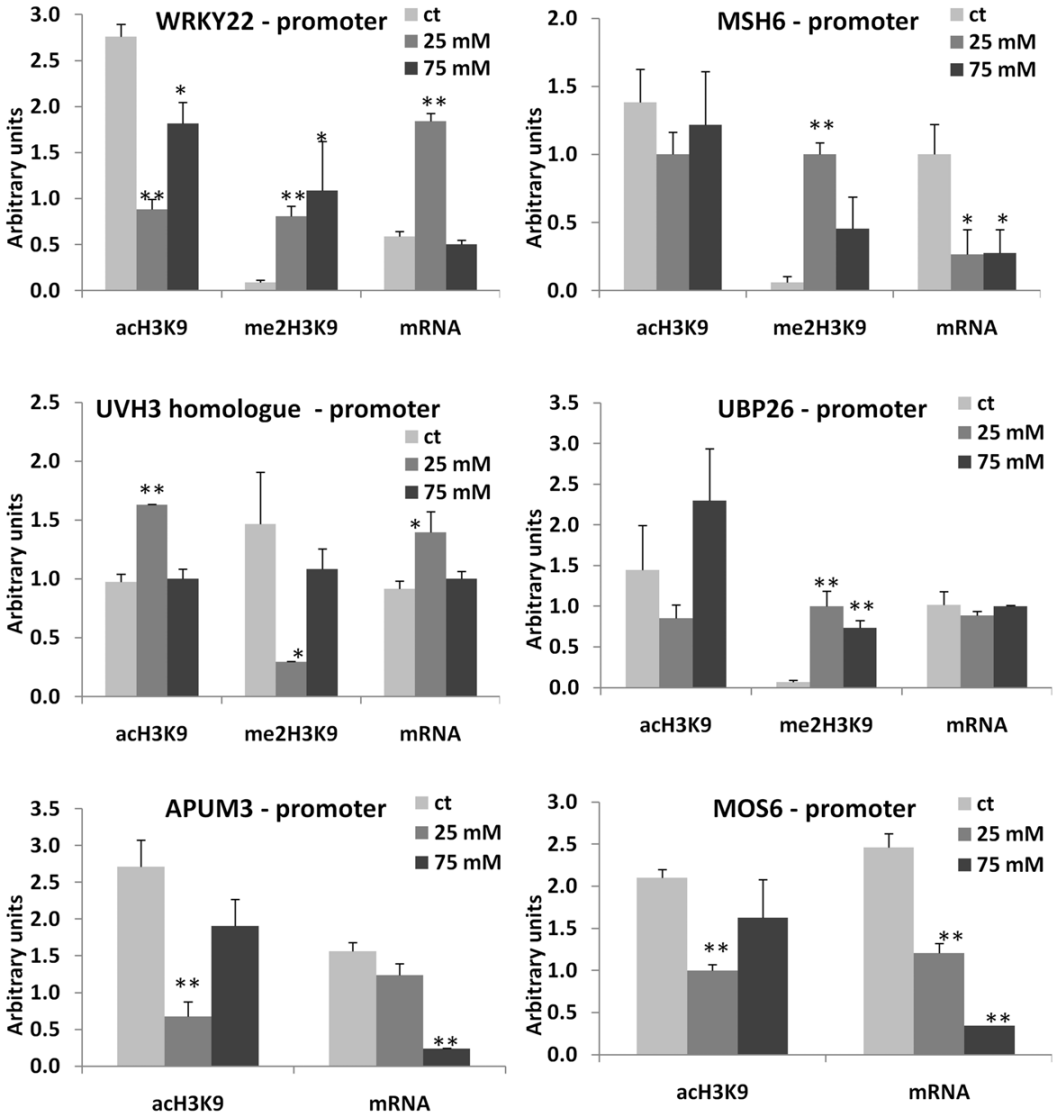
Histone modifications (H3K9me2 and H3K9ac) at the promoter regions of *WRKY22*, *MSH6*, *UHV3 homolog*, *MOS6*, *APUM3* and *UBP26* genes. The figure shows the levels of H3K9me2 and H3K9ac found at the promoter region of *WRKY22*, *MSH6*, *UVH3 homolog*, *MOS6*, *APUM3* and *UBP26* genes. Each figure also shows mRNA levels for each of the genes. The Y-axis shows the levels of mRNA expression and H3K9me2/H3K9ac in average arbitrary units (calculated from three independent experiments with SD). The asterisks denote a significant difference between the progeny of stressed (25 and 75 mM) and control plants; one asterisk stands for p<0.05 and two asterisks for p<0.01.

**Figure 6:**
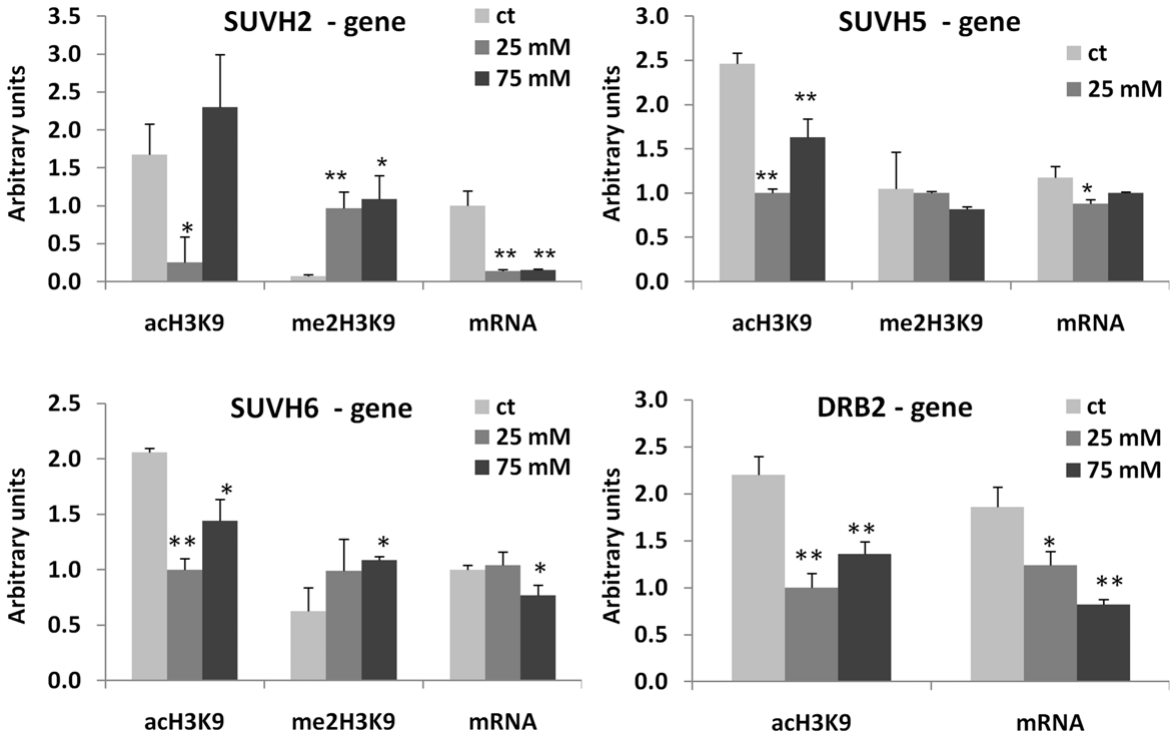
Histone modifications (H3K9me2 and H3K9ac) at the gene body regions of *SUVH2*, *SUVH5*, *SUVH6* and *DRB2* genes. The figure shows the levels of H3K9me2 and H3K9ac found at the gene body regions of *SUVH2*, *SUVH5*, *SUVH6* and *DRB2* genes. Each figure also shows mRNA levels for each of the genes. The Y-axis shows the levels of mRNA expression and H3K9me2/H3K9ac in average arbitrary units (calculated from three independent experiments with SD). The asterisks denote a significant difference between the progeny of stressed (25 and 75 mM) and control plants; one asterisk stands for p<0.05 and two asterisks for p<0.01.

**Figure 7:**
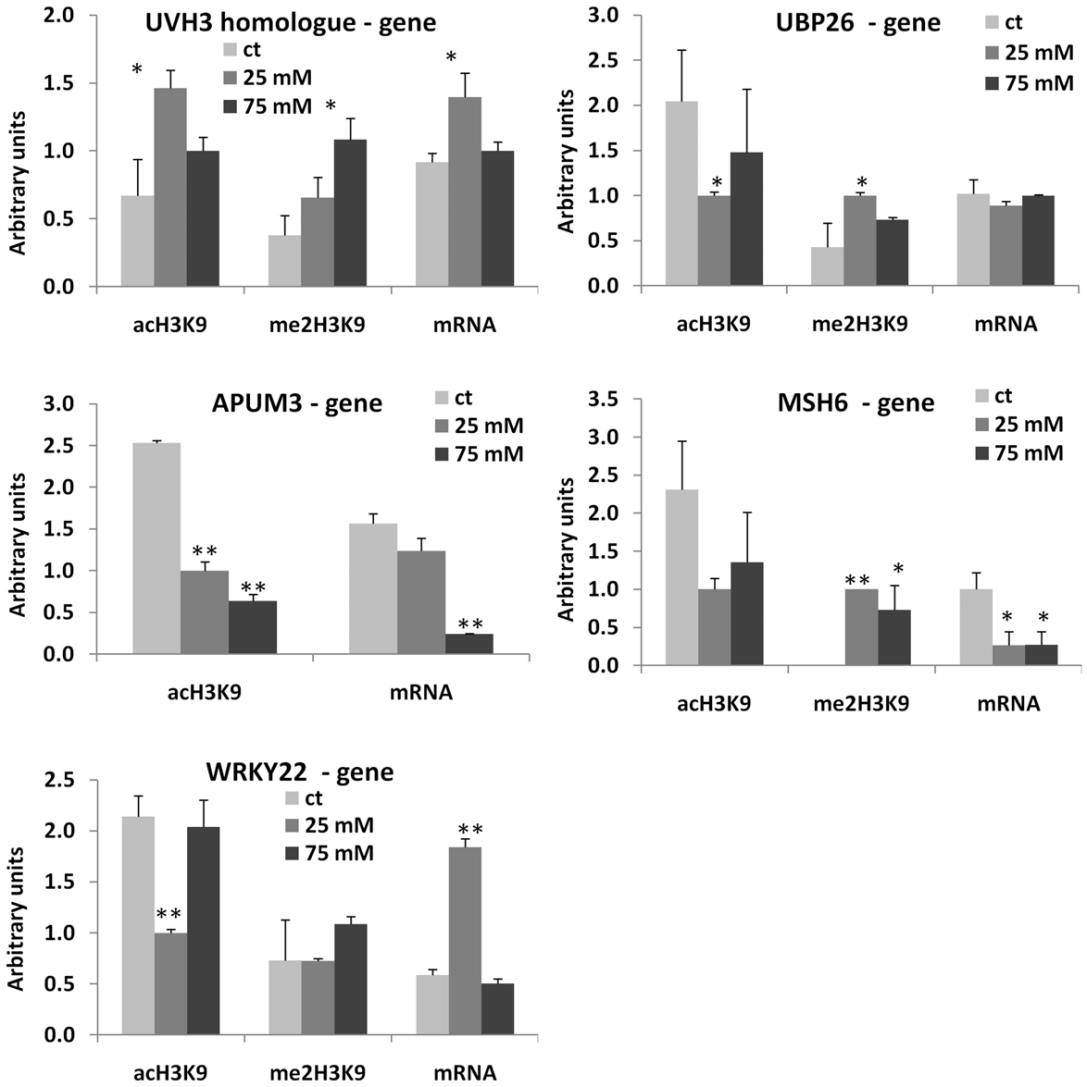
Histone modifications (H3K9me2 and H3K9ac) at the gene body regions of *UBP26*, *WRKY22*, *MSH6*, *UHV3 homolog* and *APUM3* genes. The figure shows the levels H3K9me2 and H3K9ac observed at the gene body regions of *WRKY22*, *MSH6*, *UVH3 homolog*, *MOS6*, *APUM3* and *UBP26* genes. Each figure also shows mRNA levels for each of the genes. The Y-axis shows the levels of mRNA expression and H3K9me2/H3K9ac in average arbitrary units (calculated from three independent experiments with SD). The asterisks denote a significant difference between the progeny of stressed (25 and 75 mM) and control plants; one asterisk stands for p<0.05 and two asterisks for p<0.01.

Being a part of transcription regulation process, DNA methylation often correlates with specific histone modifications. Specifically, the promoter regions correlate with H3K9ac, whereas the transcribed regions correlate with H3K9me2 [9]. We hypothesized that hypermethylated promoters in the progeny of stressed plants should have a lower level of H3K9ac and a higher level of H3K9me2. To test this hypothesis, we chose 12 genes from which 7 (*SUVH5*, *SUVH6*, *SUH8*, *ROS1*, *MOS6*, *WRKY22*, *MSH6*) were hypermethylated at the promoter region, four genes (*SUVH2*, *UBP26*, *DRB2*, *APUM3*) were hypermethylated at the transcribed region, and one gene (*UVH3* homolog) was hypomethylated at the promoter region of at least one of the progenies of stressed plants (exposed to 25 or 75 mM NaCl). To analyze histone modifications associated with specific genomic regions, we performed the chromatin immunoprecipitation assay using anti-H3K9ac and anti-H3K9me2 antibodies followed by the quantitative PCR (ChIP-qPCR) analysis using both promoter- and gene-specific primers (Table S4). In the majority of the cases, we indeed found that the hypermethylated promoters were associated with a decrease in the level of H3K9ac (r = −0.6 on average, except for *SUVH8* and *WRKY22*) and an increase in the level of H3K9me2 (r = 0.6 on average) in the progeny of stressed plants (Figure 4, 5, 6, 7; Table S5). The transcribed regions of these genes were also associated with similar histone modifications; methylation at the promoter region correlated negatively with H3K9ac (r = −0.5 on average) and correlated positively with H3K9me2 (r = 0.6). The *SUVH2*, *UBP26*, *DRB2*, *APUM3* genes that were found to be hypermethylated at gene bodies were also shown to have a lower level of H3K9ac and a higher level of H3K9me2 in both promoter and gene-body regions. The level of methylation correlated negatively with H3K9ac (r = −0.5 in promoter and gene-body regions) and correlated positively with H3K9me2 (r = 0.7 and r = 0.8 in promoter and gene-body regions, respectively) (Table S5). On the contrary, hypomethylation at the promoter region of a UVH3 homolog did not correlate with H3K9ac at the promoter or gene body region but negatively correlated with H3K9me2 at the gene body (r = −0.8) (Figure 5, 7). Additionally, we found a high degree of linear correlation (r = 0.8 on average) between the accumulation of H3K9ac in the promoter and transcribed regions of analyzed genes (Table S5). These experiments confirmed our hypothesis and showed a high degree of relationship between hypermethylation of the promoter or gene-body regions and the occurrence of repressive and permissive chromatin marks (Table S5, S6).

Since we found a correlation between promoter methylation and the associated chromatin marks, we hypothesized that the expression of these genes would also change in the progeny of stressed plants. The qPCR analysis indeed confirmed that in all cases, except for *WRKY22*, the genes hypermethylated at the promoter regions had lower levels of gene expression in the progeny of stressed plants (Figure 4, 5, 6, 7). The *UVH3*-like gene hypomethylated at the promoter region in the progeny of plants exposed to 25 mM NaCl showed a higher level of mRNA. The correlation analysis between levels of specific histone modifications and mRNA expression showed a positive correlation between H3K9ac and mRNA levels (r = 0.6 and r = 0.7 on average for the promoter and gene-body regions, respectively) and a negative correlation between the levels of H3K9me2 and mRNA (r = −0.7 and r = −0.5 on average for the promoter and gene-body regions, respectively) (Table S5, S6).

## Discussion

Plants exposed to stress may pass the information about it as a dominant trait on to successive generations [14]. Consequently, it can lead to an increased stress tolerance known as hardening phenomenon [2]. Such transgenerational adaptation to stress may depend on a number of epigenetic marks that mediate heritable changes in DNA methylation and chromatin structure. The dynamic modifications of the chromatin structure are essential for the correct regulation of vital nuclear processes such as DNA transcription, replication, repair, and recombination [34].

### Exposure to Stress Results in Changes in DNA Methylation in the Progeny

Somatic tissues of stressed plants may respond to stress with either a decrease or an increase in DNA methylation, depending on the genomic locus. It appears, however, that demethylation leading to the activation of gene expression is a more common immediate response to stress. In tobacco, the accumulation of several abiotic and biotic stress-induced transcripts was associated with an active demethylation process at given loci [35], [36]. Hemp and clover plants subjected to heavy metal stress also exhibited hypomethylation at several marker loci [37]. Exposure to cold stress triggered demethylation in the DNA of the nucleosome core of the *ZmMI1* gene in root tissues of maize seedlings [38]. Treatment with cold, salt and aluminum stress induced demethylation of the *NtGPDL* gene leading to higher tolerance to stress [36]. Similarly, infection of Arabidopsis plants with *Pseudomonas syringae* [39] as well as infection of tomato plants with a virus [40] triggered DNA hypomethylation at centromeric repeats and in several genomic regions involved in defence and stress responses, respectively. At the same time, *M. crystallinum* plants exposed to high salinity conditions showed a two-fold increase in CNG methylation [41]. Similarly, an age-dependent increase in methylation was sufficient to mediate resistance to the blight pathogen *X. oryzae* in rice [42].

The information about methylation changes in the progeny of stressed plants is scarce. Verhoeven et al. (2010) demonstrated that methylation changes in a population of apomictic dandelion observed upon exposure to abiotic and biotic stresses was faithfully transmitted to the progeny [43]. It was not possible, however, to deduce whether these changes were an increase or a decrease in methylation. Previously, we showed that the progeny of Arabidopsis plants exposed to different biotic [44] and abiotic [14], [45] stressors exhibited the higher frequency of homologous recombination, elevated tolerance to stress, and increased global DNA methylation.

Taking into consideration the abovementioned information, it can be hypothesized that a common response of plants to stress is demethylation of specific genomic regions followed by hypermethylation of the genome in the progeny ([2]).

Our analysis of methylation at the gene body showed that the 5′ and 3′ ends of the genes had a substantially lower level of methylation as compared to the central part of the gene. A similar distribution of methylation was also observed before. Although Cocus et al. (2008) [7] found a 5- to 8-fold difference and Lister et al. (2008) [46] observed an ∼10-fold difference between methylation levels in the central part of the gene and at either the 5′ and 3′ ends of the gene, we found a 25-fold difference in this ratio. In our work, the increase in methylation at the transcribed regions in the progeny of stressed plants was much greater at the 5′ and 3′ ends of the gene rather than at the central part of the gene. It is not clear how methylation at the 5′ or 3′ end of the gene correlates with gene expression, but it can be hypothesized that increased methylation at these regions of the gene would negatively impact gene expression.

Another interesting result of our studies was the difference in methylation levels between exons and introns. We found that the level of methylation in exons was higher than that in introns. However, and it was more important that the progeny of stressed plants had a higher increase in methylation in exons than in introns. As it is suggested by Feng and Jacobsen (2011), it is not clear what the role of methylation at gene bodies is since the expression of most of the genes does not change with a decrease in methylation at the gene body observed in mutants impaired in DNA methylation [9]. It is proposed that methylation may regulate exon definition or/and splicing controlling the production of alternative transcripts [9]. We hypothesize that an increase in methylation in exons in the progeny of stressed plants may control transcription, splicing or perhaps, the potential rearrangements, thus preventing reshuffling of exons. It remains to be shown whether the number of alternative transcripts and their frequency of occurrence decrease in the progeny of stressed plants. However, it should be noted that in our experiments, we used C24 plants and the analysis of methylation was performed on microarrays that are based on DNA sequences from the Columbia ecotype, therefore, some of the changes in methylation may have either been over- or under-represented.

### Changes in DNA Methylation in the Progeny of Stressed Plants Correlate with Changes in Histone Modifications

Changing DNA methylation is not the only way to epigenetically control gene expression in response to stress. It was recently demonstrated that activation of repetitive elements in heat-stressed Arabidopsis plants occurs without loss of DNA methylation but rather due to heterochromatin decondensation and nucleosome loss [47]. Changes in histone modifications were shown to be solely responsible for reactivation of silenced transgenes; exposure to several different abiotic stresses resulted in a release of transgene silencing without loss of DNA methylation via altering histone occupancy and inducing histone H3 acetylation [23].

The level of DNA methylation frequently affects gene expression together with changes in histone code [48]. For instance, dimethylation of lysine 9 and lysine 27 of histone H3 (H3K9me2, H3K27me2) in plants [49] together with hypermethylation of DNA are linked to the transcriptional repression, while dimethylation of lysine 4 and/or acetylation of lysine 9 of histone H3 (H3K4me2, H3K9ac) and hypomethylation of DNA of the promoter region are associated with an active gene. We attempted to find out whether changes in DNA methylation in the progeny of stressed plants are also paralleled by changes in histone modifications. Using the chromatin immunoprecipitation method (ChIP), we found a positive correlation between the level of DNA methylation and the occurrence of the repressive chromatin H3K9me2 mark in the progenies of stressed plants. Additionally, a high level of H3K9me2 at a chosen DNA locus was paralleled by a decreased level of H3K9ac and gene expression. Until now, no data on changes in the level of H3K9ac or H3K9me2 in the progeny of stressed plants exist, however, changes in H3K9 modifications in stressed somatic tissues are well documented. Exposure to drought resulted in an increase in histone acetylation in the promoters of stress-induced genes [50]. Also, exposure to UV-B triggered increase in histone acetylation in Arabidopsis plants and wheat [51], [52]. Similarly, Lang-Mladek (2010) showed that temperature and UV-B resulted in histone acetylation of a silent reporter gene [23]. Unfortunately, no information on changes in the progeny of these plants was provided.

H3K9 methylation in Arabidopsis plants is maintained by SET-domain proteins, including KRYPTONITE/SUVH4 (KYP/SUVH4), SUVH5, SUVH6 and SUVH2 [53], [54]. The *kyp* mutations cause a decrease in H3K9 methylation, loss of CNG DNA methylation, and reduced gene silencing [54]. A similar correlation between DNA and histone methylation was shown in studies of *Neurospora crassa* [55], further suggesting that H3K9 methylation is tightly linked to DNA methylation in different species.

In our studies, the Arabidopsis SU(VAR)3-9 homologs, namely *SUVH5*, *SUVH6*, *SUVH8*, were hypermethylated in the promoter regions and *SUVH2* - in the coding regions in the progenies of salt-stressed plants. The expression analysis showed a decrease in the level of mRNA in these genes regardless of the fact that methylation changes were observed either in the promoters or gene bodies. It is possible that hypermethylation of these homologs may represent a protective mechanism against hypermethylation of the genome in the progeny of stressed plants. Recently, it was shown that *suvh2* mutant as well as mutants impaired in siRNA biogenesis exhibited increased rate of ONSEN activation when exposed to heat stress [56]. Thus, decrease in the expression of SU(VAR)3-9 homologs may contribute to transposon activation.

Several other genes involved in either DNA repair or chromatin modifications showed altered methylation in the progeny of stressed plants, including *UBP26*, *MSH6* and *ROS1*. UBP26 protein facilitates heterochromatin formation by removing ubiquitin modifications of histone H2B; therefore, it is vital for endosperm development and flowering [29]. It can also be hypothesized that hypermethylation of *UBP26* with the decrease of its expression levels may lead to local euchromatization events. Being part of the MutSα heterodimer complex, a mismatch repair protein, MSH6, together with MSH2 are involved in the initial recognition of DNA errors [57]. Our analysis showed an inverse correlation between the level of repressive chromatin marks and expression of the *MSH6* gene. Reduced expression of mismatch repair genes followed by lower levels of mismatch repair activities may result in a higher frequency of point mutations and, possibly, other genomic rearrangements in the progeny of stressed plants. Indeed, that is exactly what was observed in the progeny of plants exposed to various stresses [15], [58], [59].

*ROS1* gene encodes a member of the DEMETER (DME) family of DNA glycosylases that catalyzes the excision of methylated cytosines, thereby antagonizing the activity of DNA methyltransferases [31], [53]. The ChIP and qRTPCR analysis of this gene showed enrichment of the repressive chromatin mark H3K9me2 in both the promoter and coding regions paralleled by a slight depletion of mRNA levels and a decrease in the permissive chromatin mark H3K9ac. The decrease in *ROS1* expression may result in a lower ability of repairing DNA as well as removing methylated cytosines.Loss of the *ROS1* gene induces hypermethylation of cytosine residues within plant-specific CNG sequences [60] and transcriptional silencing of transgenes, endogenous genes, and transposon sequences [61]. These results are consistent with our data which show an increase in methylation of transposons in the progeny of salt-stressed plants [14].

The exact reason for *ROS1* transcriptional repression in the progeny of stressed plants is unknown. Possibly, in order to avoid demethylation of hypermethylated loci, the *ROS1* gene is partially silenced by the repressive chromatin marks. This effect can be related to the ROS1-mediated compensatory mechanism that has been shown to exist between the PolIV/RDR2/DCL3/AGO4 pathway and *ROS1* gene expression. This pathway is responsible for RNA – dependent DNA methylation (RdDM) in Arabidopsis and is required for *de novo* DNA methylation by the methyltransferase DOMAINS REARRANGED METHYLTRANSFERASE2 (DRM2) as well as for the maintenance of non-CG methylation by CMT3. It was observed that in *rdr2* and *drm2* mutant plants, genes that are normally demethylated by ROS1 accumulated CG and non-CG methylation. The authors speculated that DNA hypermethylation was due to the *ROS1* down-regulation occurred in these mutants [61]. Also, SUVH5 was suggested as a possible candidate that could mediate non-CG DNA methylation through CMT3 activity [28]. Therefore, silencing of the members of the HMTase family can possibly mediate down-regulation of *ROS1* expression through the PolIV/RDR2/DCL3/AGO4 pathway. The fact that we observed reduced expression of HMTases and *ROS1*, in part, supports this hypothesis. The future analysis of the chromatin marks of the ROS1- target loci in the *suvh* mutants may reveal a possible link between HMTases and DME proteins.

The exact mechanism of hypermethylation of specific genomic loci coding for chromatin modifiers in the progeny of stressed plants is still unknown [1]. Exposure to stress may result in the accumulation of specific siRNAs triggering *de novo* RdDM at non-CG sites in addition to programmed changes in methylation at symmetrical cytosines [62]. Thus, one of the possible directions that need to be explored to clarify the inheritance of epigenetic marks in stressed plants is RNA-dependent DNA methylation. Of note is the fact that the analysis of methylation among genes involved in small RNA biogenesis showed that *DCL2*, *DCL3* and *DRD3* were equally methylated in the progeny of control and stressed plants, whereas *DRD2*, *DDL*, *AGO6* were slightly hypermethylated in the progeny of stressed plants (Table 6). The future analysis of the global small RNA profiles with relation to potential genome targets for methylation and histone modifications in the progeny of stressed plants will allow better understanding of the mechanism of epigenetic transgenerational memory.

**Table 6:** Methylation in genes encoding proteins involved in siRNA biogenesis.

Gene number	Gene name	Methylation
AT3G03300	DCL2	equally methylated
AT3G20550	DDL	slightly hypermethylated in 25 and 75 mM
AT3G43920	DCL3	equally methylated
AT2G32940	AGO6	slightly hypermethylated in 25 and 75 mM
AT3G23780	DRD2 (NRPD2)	slightly hypermethylated in 25 mM
AT2G40030	DRD3	equally methylated

## Materials and Methods

### An Experimental Set-up

In order to check the effect of NaCl stress, the Arabidopsis plants from line 11 (ecotype C24) [63], [64] were germinated and grown for three weeks on sterile MS media supplemented with either 0, 25 or 75 mM NaCl. Then, the plants were transferred into soil and grown at 22°C under 12 h day/12 h night conditions and illumination at 100 µM m^−2^ sec^−1^. In every case, seeds from 20 plants were pooled together, and plants were propagated to the next generation under normal growth conditions. The seeds were germinated and grown on soil at 22°C under 12 h day/12 h night conditions and illumination at 100 µM m^−2^ sec^−1^. Tissue samples (leaves only) from these plants were harvested at three weeks after germination and were used for further analysis.

### Immunoprecipitation Analysis of Methylated DNA

The Methyl-DNA immunoprecipitation (MeDIP) assay was performed to analyse DNA methylation [5]. Genomic DNA used for the analysis was prepared from 20 three-week-old progeny of salt-stressed *A. thaliana* plants using a Trizol reagent as published before [65]. DNA was sheared by sonication to 500- to 1,500-bp fragments followed by immunoprecipitation with antibodies against methylated cytosine [5]. 500 ng of control DNA and the entire immunoprecipitation reaction were amplified using the T7 RNA polymerase linear amplification protocol as described [5]. Control and immunoprecipitated DNA were labelled with Cy3 and Cy5 fluorescent dyes, respectively.

Both samples with labelled DNA were hybridized to Whole Genome Tiling Array 2 (Cat. # C4348001-02-01, NimbleGen). The Array 2 contains probes, 90-nt long, covering the entire DNA sequence of chromosome 3 and the partial DNA sequences of chromosomes 2 and 4. The sequence of chromosome 2 consists of the region from nt position 9,687,916 to the end of chromosome at nt position 19,704,755. The sequence of chromosome 4 consists of the region from nt position 1,001 to nt position 6,133,069.

For the data normalization (performed by the Tukey-biweight scaling procedure) and statistical analysis, we used the R environment including the package Ringo [66]. Furthermore, for the identification of the ChIP-enriched regions, we followed an overall description made by Toedling and co-workers [66]. After the preprocessing step we did a smoothing over individual probe intensities. We performed a sliding windows procedure (with 900 bp width) along the chromosomes and replaced the intensity at each genomic position by the median over the intensities of those reporters inside the window that is centered at this position. Next, we identified the ChIP-enriched regions by taking into account that the region should contain at least three probe match positions and that the smoothed intensities of the reporters mapped to those regions exceed a defined threshold. This threshold is an upper bound for values arising from the underlying null distribution (the levels of smoothed reporters follow a mixture of two distributions, the null distribution of non-affected reporters and the alternative distribution for the values in the ChIP-enriched regions), thus smoothed probe levels larger than defined threshold are more likely to arise from the alternative (ChIP enrichment) distribution and are taken as indicator for finding ChIP-enriched regions.

Array intensities for the MeDIP analysis were represented as log_2_ signal ratios of immunoprecipitated DNA to input DNA.

A more detailed analysis of methylation was done by using either 5- or 1-kb sequence of the promoter region and the coding sequence itself. The log2 ratio IP/INPUT values of individual reporters were taken into consideration for the analysis of the number of methylated reporters that are different between groups (“ct”, “25” and “75”). Genomic regions (promoter and coding sequences) in which at least 5 reporters had different log2 IP/INPUT ratios between “75” and “ct” as well as “25” and “ct” were then taken into consideration. First the percentage of methylated reporters in each group was calculated. Next, the percentages of methylated reporters were intercompared for aforementioned groups and genomic regions in which the differences were over 50% for promoter regions and over 80% for coding sequence regions were short-listed (24 promoters and 22 coding region sequences).

To identify whether methylated regions in the “ct”, “25 mM” and “75 mM” plants groups have different values, we performed the non-parametric statistical Wilcoxon rank-sum test. At first, we ranked all values in each array (ct, 25 mM, and 75 mM) and extracted a 1 percent tail on the right-hand side (high methylation) and the left-hand side (low methylation) followed by ranking the corresponding values of other arrays. The differences between “25” and “ct”, “75” and “ct”, and “75” and “25” were expressed in p-values. A separate analysis was performed for promoter regions (4584 regions), gene body regions (2179 regions) and all regions (6763 regions) (Table S1). A similar analysis was performed for 0.1%, 0.5%, 5.0%, 10.0%, 15.0% and 20.0% tails (Table S2).

### ChIP-qPCR Analysis

All procedures for the ChIP analysis of histone modifications in the progeny of salt-stressed Arabidopsis plants were done according to the protocol described before [67] with minor modifications. Instead of using Salmon sperm DNA/protein A agarose beads, we found more convenient to use Protein G MagneticBeads (GenScript, cat.# - L002274). For immunoprecipitation, we used ChIP grade antibodies against acetyl H3K9 (Millipore, cat. # - 17-658) and methyl 2 H3K9 (Abcam, cat. # - ab1220). A no-antibody negative control was performed to measure the non-specific binding of DNA to the Protein G MagneticBeads (Figure S4). All quantitative measurements of precipitated DNA were performed using the qPCR technique with SsoFast EvaGreen Supermix (Bio-Rad, cat. #1725200) using either the promoter- or gene- specific primers. The promoter and transcribed region sequences were analyzed using EMBOSS CpGPlot software with a default settings in order to identify (if it was possible) and plot CpG islands that were used for further amplification (Figure S5) [68]. Primers for the real-time quantitative PCR were designed using the Beacon Designer7 software (Table S4). The precipitated gDNA fragments were amplified under the following conditions: (1) 98°C for 2 min for one cycle; 98°C for 5 s, 48°C for 5 s, for 40 cycles; (2) melt-curve analysis – 65°C to 95°C for 5 s, with a 0.5°C increment. The optimization of the annealing temperature, melt-curve analysis, and gel analysis of amplicons were performed for each set of primers. The normalization was done against ACTIN7 (AT5G09810). The average of four reactions (two dilutions per each of two DNA preparations stemming from two independent experiments) was obtained, and the normalized expression ratio was calculated using 2^−ΔΔCT^ method.

### MeDIP-qPCR

DNA precipitated through MeDIP was used for real time PCR of *SUVH2*, *SUVH6*, *WRKY22*, *MSH6*, *UBP26* and *UHV3* homolog genes. PCRs were performed as in “Chip-qPCR”. Primers are listed in Table S4.

### Real-Time qPCR Analysis

Approximately 80 mg of plant tissue was ground in liquid nitrogen and transferred to a chilled 1.5-mL Eppendorf tube, and 160 µL of TRIzol reagent (Invitrogen) was added. The remainder of the extraction was performed as per the manufacturer's protocol. Next, mRNA was purified and concentrated using the Oligotex mRNA Mini Kit (Qiagen, cat. # 70022). The quantity and quality of mRNA were measured in RNase-free double distilled water using a spectrophotometer. cDNA was then prepared from mRNA using the iScript Select cDNA synthesis kit (Bio-Rad, cat. # 170-8897) according to the manufacturer's protocol.

The real-time quantitative PCR was performed using SsoFast EvaGreen Supermix (Bio-Rad). cDNAs were amplified under the following conditions: (1) 98°C for 2 min for one cycle; 98°C for 5 s, 48°C for 5 s, 65°C to 95°C for 5 s; for 40 cycles; (2) melt-curve analysis - 65°C to 95°C for 5 s, with a 0.5°C increment. Primers for the real-time quantitative PCR were designed using the Beacon Designer7 program (Table S4). The optimization of the annealing temperature, melt-curve analysis, and gel analysis of amplicons were performed for each set of primers. To evaluate the PCR efficiency, the standard curve was established using a series of cDNA dilutions. The expression of genes was related to the expression of RCE1 and tubulin. The average of four reactions (two independent experiments in two technical replicates) was obtained, and the normalized expression ratio was calculated using 2^−ΔΔCT^ method.

### Statistical Treatment of the Data

Statistical analyses were performed using MS Excel software and Microcal Origin 6.0. Standard errors or standard deviations were calculated. A statistically significant difference between the means was compared using either Student's t-test or single-factor ANOVA. Statistical analysis of the percentage of non-TE genes with differentially methylated regions was performed using single-factor ANOVA; since no replication of methylation analysis was performed, statistical analysis was performed by comparing the percentage of hyper- and hypo-methylated regions in 25 mM and 75 mM plant groups (Figure 2). Statistical analysis of the percentage of TE-genes was performed between either hyper- or hypo-methylation groups and control group using data for both 25 and 75 mM plant groups (single-factor ANOVA).

## Supporting Information

Figure S1 **A list of genes that are hypermethylated or hypomethylated** ***at the promoter regions*** **in the progeny of plants exposed to 25 and 75 mM NaCl as compared to the progeny of control plants.** The file consists of the following comparison groups: “25-CT_Hyper” – hypermethylated genes in “25 mM” plants; “25-CT_Hypo” – hypomethylated genes in “25 mM” plants; “75-CT_Hyper” – hypermethylated genes in “75 mM” plants; “75-CT_Hypo” – shows hypomethylated genes in “75 mM” plants. Each list consists only of genes in which methylation changes accounted for more than 50%. “sig. reporters” indicates the number of methylated reporters in ct, 25 and 75 mM plant groups.
(XLSX)

Figure S2 **A list of genes that are hypermethylated or hypomethylated** ***at the gene body*** **in the progeny of plants exposed to 25 and 75 mM NaCl as compared to the progeny of control plants.** The file consists of the following comparison groups: “25-CT_Hyper” – hypermethylated genes in “25 mM” plants; “25-CT_Hypo” – shows hypomethylated genes in “25 mM” plants; “75-CT_Hyper” – hypermethylated genes in “75 mM” plants; “75-CT_Hypo” – hypomethylated genes in “75 mM” plants. Each list consists only of genes in which methylation changes accounted for more than 50%. “sig. reporters” –indicates the number of methylated reporters in ct, 25 and 75 mM plant groups.
(XLSX)

Figure S3 **Analysis of methylation at the promoter and gene body regions of** ***SUVH2*** **,** ***SUVH6*** **,** ***WRKY22*** **,** ***MSH6*** **,** ***UBP26*** **and** ***UVH3*** **homolog genes as measured by MeDIP-qPCR.** The Y-axis shows the methylation levels in average arbitrary units (calculated from two independent biological repeats and two technical repeats with SEM). The asterisks denote a significant difference between the progeny of stressed (25 and 75 mM) and control plants; one asterisk stands for p<0.05, two asterisks for p<0.01 and three for p<0.001 (Student's t-test).
(TIF)

Figure S4 **Representative pictures of the amplification of ACTIN7 from the H3K9ac immunoprecipitated DNA.** Amplification from DNA immunoprecipitated without antibodies. A. Amplification of the ACTIN7 gene fragment from DNA immunoprecipitated using antibodies against H3K9ac. B. Amplification of the ACTIN7 gene fragment from DNA immunoprecipitated without antibodies. No amplification was observed in over 45 cycles.
(TIF)

Figure S5 **Schematic representation of the promoter and transcribed regions of the genes used in this study.** CpG islands were plotted (where it was applicable) using EMBOSS CpGPlot software with default settings. The annealing positions of primers used for ChIP-qPCR and RT-qPCR analysis are shown for each gene.
(TIF)

Table S1 Summary of statistical analysis of differences in DNA methylation - the non-parametric statistical Wilcoxon rank-sum test. The values in each array (ct, 25 mM, and 75 mM) were ranked, and a 1.0% tail was extracted for either the left-hand side (start, low methylation) or the right-hand side (end, high methylation). In each case, ranking the corresponding values in other arrays was also performed. The differences between “25” and “ct”, “75” and “ct”, and “75” and “25” were expressed in p-values. Insignificant differences are in bold.
(DOCX)

Table S2 Summary of statistical analysis of differences in DNA methylation - the non-parametric statistical Wilcoxon rank-sum test. The values in each array (ct, 25 mM, and 75 mM) were ranked; and 0.1%, 0.5%, 1.0%, 5.0%, 10.0%, 15.0% and 20.0% tails were extracted on the left-hand side (the start, low methylation) and the right-hand side (the end, high methylation). In each case, ranking the corresponding values of other arrays was also performed. The differences between “25” and “ct”, “75” and “ct”, and “75” and “25” were expressed in p-values.
(DOCX)

Table S3 Sequence polymorphism between C24 and Columbia genomes. TAIR database was used for the analysis of sequence polymorphism. Table shows the gene ID, gene symbol, number of polymorphisms in each gene, type of substitutions, gene length and percentage of polymorphism.
(DOCX)

Table S4 Primers used for the analysis of histone modifications and gene expression. “ChiP primers” – primers used for amplification of immunoprecipitated DNA. ‘qPCR” – primers used for the analysis of gene expression. “SUVH6-p-for” and “SUVH6-p-rev” – forward and reverse primers used for amplification of promoter regions. “SUVH6-t-for” and “SUVH6-t-rev” – forward and reverse primers used for amplification of the transcript (coding) regions.
(DOCX)

Table S5 Correlation analysis performed between the following parameters: “prom-H3K9Ac”, “prom-H3K9me2”, “gene-H3K9Ac”, “gene-H3K9me2”, ‘mRNA”.
(DOCX)

Table S6 Comparison of methylation, gene expression and histone modifications data. “Number” – the gene number; “Promoter/gene” – the region analyzed; “Symbol” – a gene symbol; “methylation” – the region in which methylation difference between the progeny of stressed and control plants was identified (“Promoter 75 hyper” – means that the progeny of plants exposed to 75 mM NaCl were hypermethylated at the promoter region); “expression” – the mRNA level in the progeny of plants exposed to either 25 mM or 75 mM NaCl as compared to the progeny of control plants, “−”– the lower level, “+”– the higher level, “ = ”– a similar level of expression; “H3K9ac” and “H3K9me2” – the level of specific modification in the promoter and gene body regions in the progeny of stressed plants compared to the progeny of control plants.
(DOCX)
