# Supplementary figures and images for: The Progeny of Arabidopsis thaliana Plants Exposed to Salt Exhibit Changes in DNA Methylation, Histone Modifications and Gene Expression
Source: PLoS One. 2012 Jan 23;7(1):e30515. doi: 10.1371/journal.pone.0030515 (PMC3264603; doi:10.1371/journal.pone.0030515)

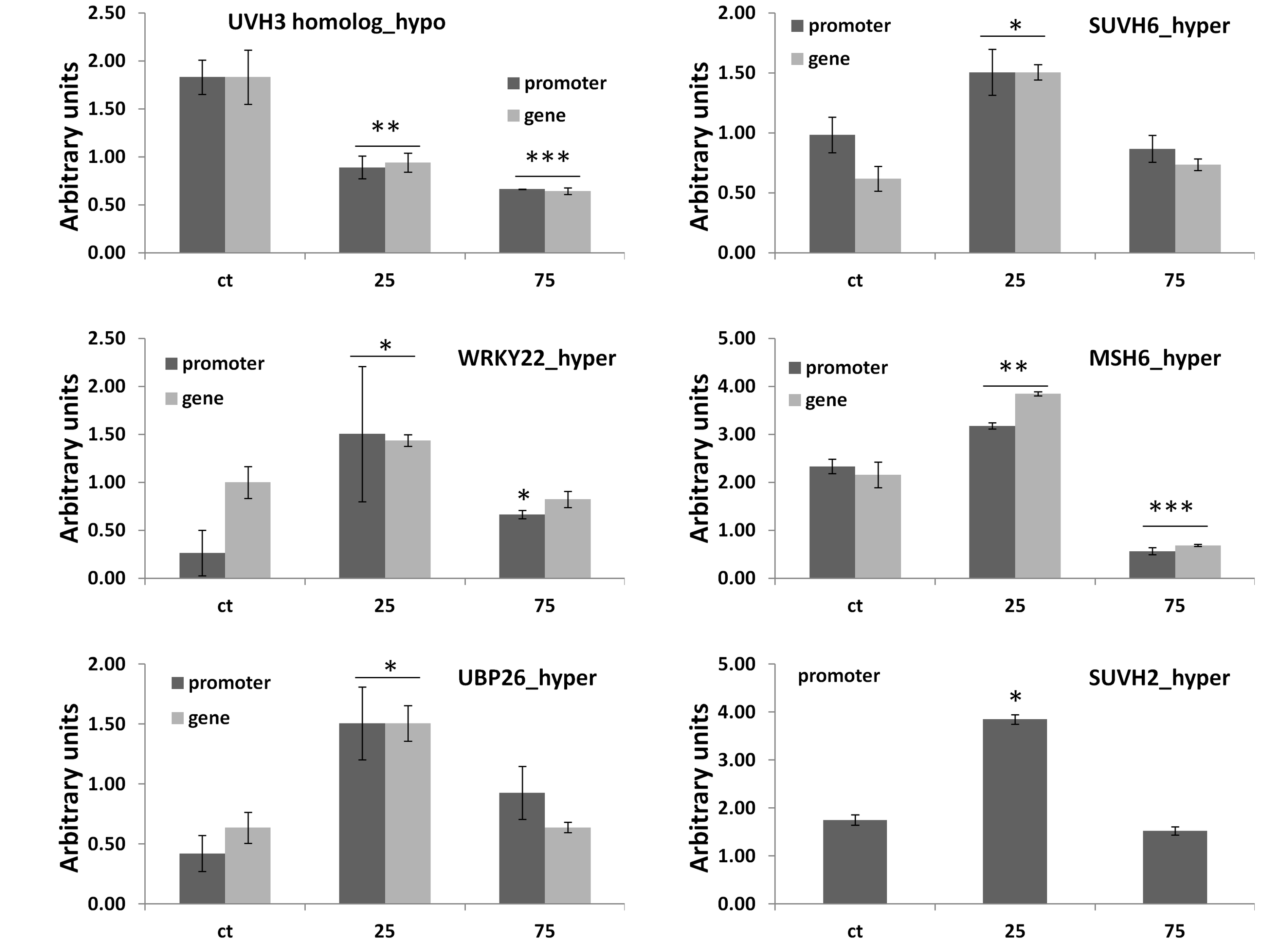

Supplement: Figure S3 — Analysis of methylation at the promoter and gene body regions of SUVH2 , SUVH6 , WRKY22 , MSH6 , UBP26 and UVH3 homolog genes as measured by MeDIP-qPCR. The Y-axis shows the methylation levels in average arbitrary units (calculated from two independent biological repeats and two technical repeats with SEM). The asterisks denote a significant difference between the progeny of stressed (25 and 75 mM) and control plants; one asterisk stands for p<0.05, two asterisks for p<0.01 and three for p<0.001 (Student's t-test). (TIF) [file pone.0030515.s003.tif]

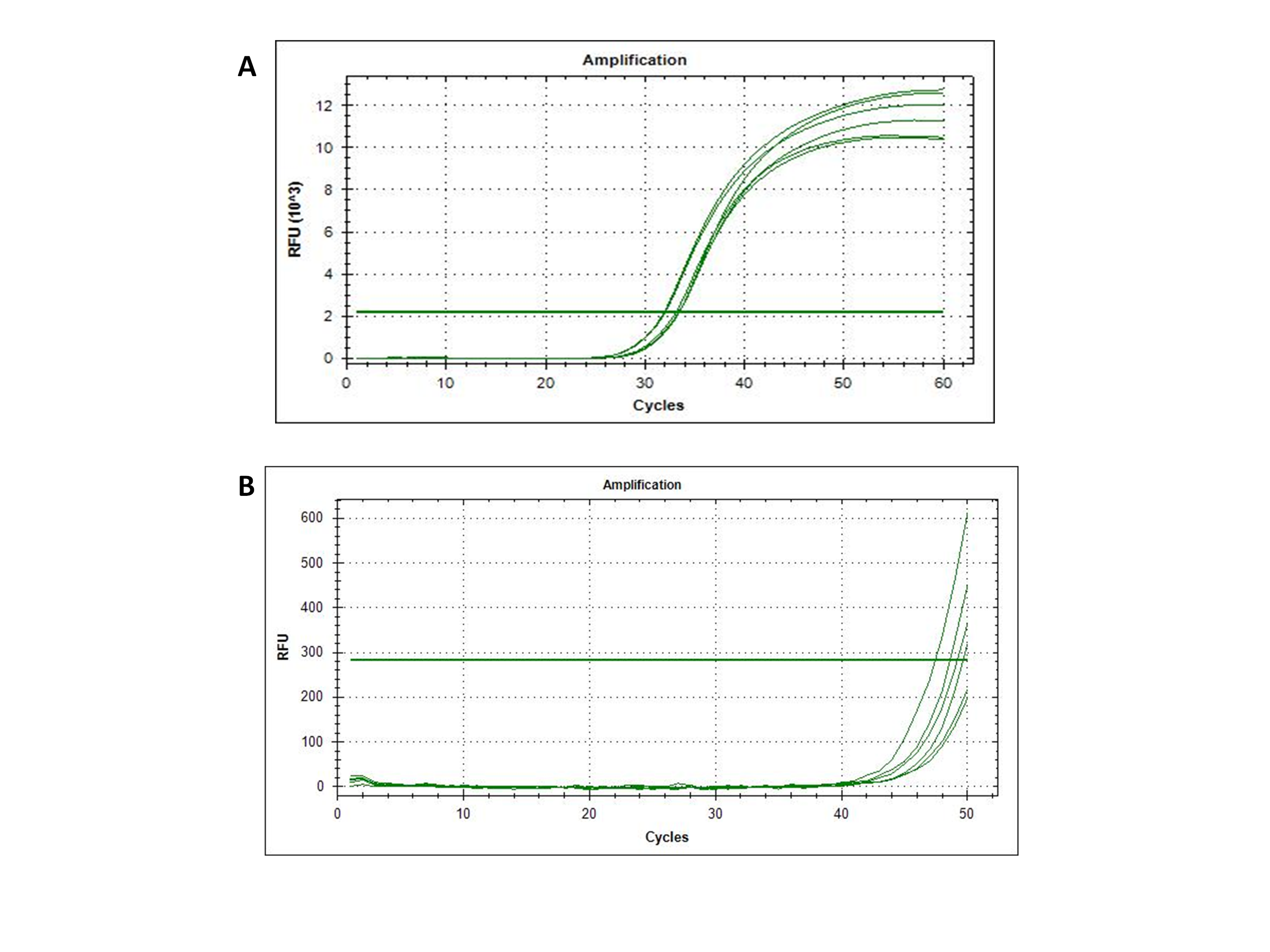

Supplement: Figure S4 — Representative pictures of the amplification of ACTIN7 from the H3K9ac immunoprecipitated DNA. Amplification from DNA immunoprecipitated without antibodies. A. Amplification of the ACTIN7 gene fragment from DNA immunoprecipitated using antibodies against H3K9ac. B. Amplification of the ACTIN7 gene fragment from DNA immunoprecipitated without antibodies. No amplification was observed in over 45 cycles. (TIF) [file pone.0030515.s004.tif]

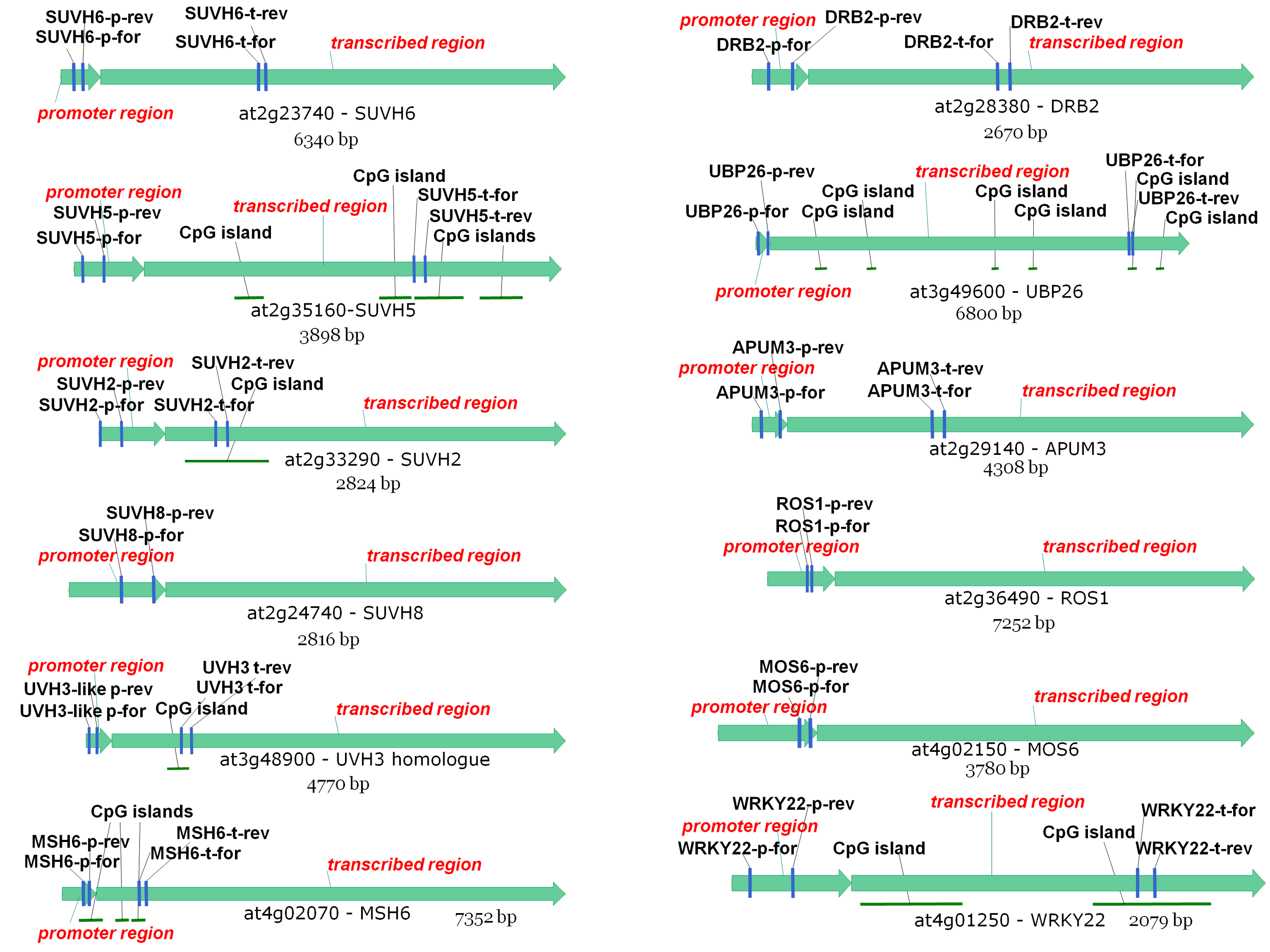

Supplement: Figure S5 — Schematic representation of the promoter and transcribed regions of the genes used in this study. CpG islands were plotted (where it was applicable) using EMBOSS CpGPlot software with default settings. The annealing positions of primers used for ChIP-qPCR and RT-qPCR analysis are shown for each gene. (TIF) [file pone.0030515.s005.tif]
