# Supplementary material for: The Progeny of Arabidopsis thaliana Plants Exposed to Salt Exhibit Changes in DNA Methylation, Histone Modifications and Gene Expression
Source: PLoS One. 2012 Jan 23;7(1):e30515. doi: 10.1371/journal.pone.0030515 (PMC3264603; doi:10.1371/journal.pone.0030515)
Supplement: Table S1 — Summary of statistical analysis of differences in DNA methylation - the non-parametric statistical Wilcoxon rank-sum test. The values in each array (ct, 25 mM, and 75 mM) were ranked, and a 1.0% tail was extracted for either the left-hand side (start, low methylation) or the right-hand side (end, high methylation). In each case, ranking the corresponding values in other arrays was also performed. The differences between “25” and “ct”, “75” and “ct”, and “75” and “25” were expressed in p-values. Insignificant differences are in bold. (DOCX) [file pone.0030515.s006.docx]

**Table S1. Summary of statistical analysis of differences in DNA methylation - the non-parametric statistical Wilcoxon rank-sum test**

|  | **Promoter region** | | | **Gene-body region** | | | **All regions** | | |
| --- | --- | --- | --- | --- | --- | --- | --- | --- | --- |
|  | **ct vs 25** | **ct vs 75** | **25 vs 75** | **ct vs 25** | **ct vs 75** | **25 vs 75** | **ct vs 25** | **ct vs 75** | **25 vs 75** |
| **ct 1.0% tail (the start)** | **1.00E+00** | 7.01E-19 | 7.01E-19 | **1.00E+00** | 1.54E-09 | 1.54E-09 | **1.00E+00** | 3.88E-27 | 3.88E-27 |
| **ct 1.0% tail (the end)** | 7.25E-10 | 1.86E-09 | **4.29E-01** | 3.85E-05 | 1.68E-07 | **3.90E-01** | 6.09E-12 | 3.17E-15 | **4.14E-01** |
| **25 1.0% tail (the start)** | **1.00E+00** | 7.01E-19 | 7.01E-19 | **1.00E+00** | 1.54E-09 | 1.54E-09 | **1.00E+00** | 3.88E-27 | 3.88E-27 |
| **25 1.0% tail (the end)** | 1.22E-06 | 7.57E-04 | 3.31E-05 | 1.63E-05 | **6.22E-02** | 1.52E-06 | 3.85E-12 | 5.53E-04 | 2.95E-13 |
| **75 1.0% tail (the start)** | 3.21E-19 | **1.00E+00** | 3.21E-19 | 1.52E-09 | **1.00E+00** | 1.52E-09 | 1.00E-27 | **1.00E+00** | 1.00E-27 |
| **75 1.0% tail (the end)** | **7.86E-02** | 4.92E-08 | 1.02E-07 | **1.00E+00** | 1.36E-09 | 1.36E-09 | **2.03E-01** | 6.11E-16 | 4.60E-16 |

The values in each array (ct, 25 mM, and 75 mM) were ranked, and a 1.0% tail was extracted for either the left-hand side (start, low methylation) or the right-hand side (end, high methylation). In each case, ranking the corresponding values in other arrays was also performed. The differences between “25” and “ct”, “75” and “ct”, and “75” and “25” were expressed in p-values. Insignificant differences are in bold.
