# Supplementary material for: The Progeny of Arabidopsis thaliana Plants Exposed to Salt Exhibit Changes in DNA Methylation, Histone Modifications and Gene Expression
Source: PLoS One. 2012 Jan 23;7(1):e30515. doi: 10.1371/journal.pone.0030515 (PMC3264603; doi:10.1371/journal.pone.0030515)
Supplement: Table S2 — Summary of statistical analysis of differences in DNA methylation - the non-parametric statistical Wilcoxon rank-sum test. The values in each array (ct, 25 mM, and 75 mM) were ranked; and 0.1%, 0.5%, 1.0%, 5.0%, 10.0%, 15.0% and 20.0% tails were extracted on the left-hand side (the start, low methylation) and the right-hand side (the end, high methylation). In each case, ranking the corresponding values of other arrays was also performed. The differences between “25” and “ct”, “75” and “ct”, and “75” and “25” were expressed in p-values. (DOCX) [file pone.0030515.s007.docx]

**Table S2. Summary of statistical analysis of difference in DNA methylation - non-parametric statistical Wilcoxon rank-sum test**

|  | **Promoter region** | | | **Gene body region** | | | **All regions** | | |
| --- | --- | --- | --- | --- | --- | --- | --- | --- | --- |
|  | **ct vs 25** | **ct vs 75** | **25 vs 75** | **ct vs 25** | **ct vs 75** | **25 vs 75** | **ct vs 25** | **ct vs 75** | **25 vs 75** |
| **ct 0.1% tail (start)** | **1.00E+00** | 8.85E-03 | 8.85E-03 | **1.00E+00** | **1.10E-01** | **1.10E-01** | **1.00E+00** | 1.13E-03 | 1.13E-03 |
| **ct 0.1% tail (end)** | 1.47E-02 | 3.98E-02 | **1.56E-01** | **9.70E-02** | **9.70E-02** | **1.00E+00** | 2.06E-03 | 3.74E-03 | **1.33E-01** |
| **25 0.1% tail (start)** | **1.00E+00** | 8.85E-03 | 8.85E-03 | **1.00E+00** | **1.10E-01** | **1.10E-01** | **1.00E+00** | 1.13E-03 | 1.13E-03 |
| **25 0.1% tail (end)** | **9.16E-02** | **1.50E-01** | **9.70E-02** | **1.10E-01** | **1.23E-01** | **1.10E-01** | 2.58E-03 | **1.10E-01** | 2.31E-03 |
| **75 0.1% tail (start)** | 6.56E-03 | **1.00E+00** | 6.56E-03 | **1.10E-01** | **1.00E+00** | **1.10E-01** | 9.10E-04 | **1.00E+00** | 9.10E-04 |
| **75 0.1% tail (end)** | **5.00E-01** | **1.44E-01** | 1.14E-02 | **1.00E+00** | **9.70E-02** | **9.70E-02** | **1.00E+00** | 6.31E-04 | 6.31E-04 |
|  |  |  |  |  |  |  |  |  |  |
| **ct 0.5% tail (start)** | **1.00E+00** | 3.62E-10 | 3.62E-10 | **1.00E+00** | 3.01E-05 | 3.01E-05 | **1.00E+00** | 2.22E-14 | 2.22E-14 |
| **ct 0.5% tail (end)** | 1.71E-06 | 1.77E-05 | 4.05E-02 | 8.65E-03 | 8.34E-05 | **4.38E-01** | 6.65E-08 | 1.79E-09 | **8.77E-02** |
| **25 0.5% tail (start)** | **1.00E+00** | 3.62E-10 | 3.62E-10 | **1.00E+00** | 3.01E-05 | 3.01E-05 | **1.00E+00** | 2.22E-14 | 2.22E-14 |
| **25 0.5% tail (end)** | 2.46E-05 | 3.34E-03 | 8.34E-05 | 2.43E-04 | **5.32E-02** | 2.64E-04 | 2.47E-07 | 1.19E-02 | 6.27E-09 |
| **75 0.5% tail (start)** | 3.69E-10 | 1.00E+00 | 3.69E-10 | 3.03E-05 | **1.00E+00** | 3.03E-05 | 2.31E-14 | **1.00E+00** | 2.31E-14 |
| **75 0.5% tail (end)** | **3.16E-01** | 1.11E-04 | 1.19E-06 | **1.00E+00** | 1.66E-05 | 1.66E-05 | **5.26E-02** | 6.27E-13 | 9.84E-12 |
|  |  |  |  |  |  |  |  |  |  |
| **ct 1.0% tail (start)** | **1.00E+00** | 7.01E-19 | 7.01E-19 | **1.00E+00** | 1.54E-09 | 1.54E-09 | **1.00E+00** | 3.88E-27 | 3.88E-27 |
| **ct 1.0% tail (end)** | 7.25E-10 | 1.86E-09 | **4.29E-01** | 3.85E-05 | 1.68E-07 | **3.90E-01** | 6.09E-12 | 3.17E-15 | **4.14E-01** |
| **25 1.0% tail (start)** | **1.00E+00** | 7.01E-19 | 7.01E-19 | **1.00E+00** | 1.54E-09 | 1.54E-09 | **1.00E+00** | 3.88E-27 | 3.88E-27 |
| **25 1.0% tail (end)** | 1.22E-06 | 7.57E-04 | 3.31E-05 | 1.63E-05 | **6.22E-02** | 1.52E-06 | 3.85E-12 | 5.53E-04 | 2.95E-13 |
| **75 1.0% tail (start)** | 3.21E-19 | **1.00E+00** | 3.21E-19 | 1.52E-09 | **1.00E+00** | 1.52E-09 | 1.00E-27 | **1.00E+00** | 1.00E-27 |
| **75 1.0% tail (end)** | **7.86E-02** | 4.92E-08 | 1.02E-07 | **1.00E+00** | 1.36E-09 | 1.36E-09 | **2.03E-01** | 6.11E-16 | 4.60E-16 |
|  |  |  |  |  |  |  |  |  |  |
| **ct 5.0% tail (start)** | **1.00E+00** | 1.38E-87 | 1.38E-87 | **1.00E+00** | 2.80E-42 | 2.80E-42 | **1.00E+00** | 3.24E-128 | 3.24E-128 |
| **ct 5.0% tail (end)** | 1.70E-27 | 2.78E-37 | **1.63E-01** | 1.01E-13 | 9.02E-25 | 3.90E-04 | 6.55E-30 | 3.21E-62 | 2.20E-05 |
| **25 5.0% tail (start)** | **1.00E+00** | 1.38E-87 | 1.38E-87 | **1.00E+00** | 2.80E-42 | 2.80E-42 | **1.00E+00** | 3.24E-128 | 3.24E-128 |
| **25 5.0% tail (end)** | 3.31E-16 | 1.38E-05 | 8.03E-16 | 2.17E-12 | **1.39E-01** | 3.90E-21 | 3.21E-19 | **1.87E-01** | 7.38E-43 |
| **75 5.0% tail (start)** | 2.17E-81 | 2.19E-03 | 5.73E-83 | 2.78E-42 | **1.00E+00** | 2.78E-42 | 3.36E-128 | **1.00E+00** | 3.36E-128 |
| **75 5.0% tail (end)** | 4.50E-05 | 1.29E-25 | 7.21E-15 | 2.97E-02 | 2.49E-15 | 1.21E-10 | 1.21E-05 | 1.60E-36 | 1.25E-19 |
|  |  |  |  |  |  |  |  |  |  |
| **ct 10% tail (start)** | **1.00E+00** | 6.55E-173 | 6.55E-173 | 1.24E-02 | 7.71E-82 | 6.37E-81 | **1.00E+00** | 3.90E-254 | 3.90E-254 |
| **ct 10.0% tail (end)** | 2.17E-45 | 1.37E-65 | **5.53E-02** | 1.17E-13 | 2.86E-41 | 3.88E-09 | 9.77E-44 | 8.16E-106 | 5.82E-09 |
| **25 10.0% tail (start)** | **1.00E+00** | 6.55E-173 | 6.55E-173 | 1.24E-02 | 2.34E-80 | 2.92E-81 | **1.00E+00** | 3.90E-254 | 3.90E-254 |
| **25 10.0% tail (end)** | 1.40E-24 | 3.54E-05 | 1.19E-29 | **6.68E-13** | **4.48E-01** | 2.43E-34 | 9.04E-35 | 3.38E-03 | 1.11E-65 |
| **75 10.0% tail (start)** | 1.54E-12 | 7.58E-70 | 2.93E-80 | 6.81E-23 | 5.52E-27 | 1.52E-53 | 1.52E-25 | 7.38E-95 | 2.99E-129 |
| **75 10.0% tail (end)** | 6.08E-06 | 9.63E-36 | 2.56E-18 | 3.79E-04 | 6.11E-20 | 7.88E-09 | 1.81E-11 | 7.35E-50 | 1.23E-16 |
|  |  |  |  |  |  |  |  |  |  |
| **ct 15.0% tail (start)** | 5.66E-45 | 4.16E-197 | 3.40E-137 | 8.74E-32 | 5.75E-95 | 4.58E-52 | 1.09E-74 | 9.51E-296 | 1.24E-187 |
| **ct 15.0% tail (end)** | 2.74E-54 | 5.66E-84 | 1.31E-02 | 4.33E-16 | 2.38E-56 | 2.92E-11 | 1.69E-60 | 3.95E-139 | 6.70E-11 |
| **25 15.0% tail (start)** | 5.49E-45 | 1.82E-118 | 1.24E-171 | 8.73E-32 | 8.41E-36 | 1.30E-72 | 1.06E-74 | 4.39E-155 | 1.21E-247 |
| **25 15.0% tail (end)** | 1.62E-31 | 3.62E-05 | 4.94E-39 | 8.69E-16 | **3.85E-01** | 1.59E-45 | 2.10E-46 | 6.30E-03 | 2.58E-83 |
| **75 15.0% tail (start)** | 1.08E-03 | 2.48E-147 | 1.93E-104 | 4.49E-05 | 1.33E-62 | 7.45E-66 | **2.56E-01** | 7.08E-208 | 7.33E-178 |
| **75 15.0% tail (end)** | 1.00E-07 | 2.76E-40 | 5.20E-19 | 5.57E-06 | 1.86E-20 | 5.07E-05 | 1.59E-12 | 1.18E-52 | 1.18E-15 |
|  |  |  |  |  |  |  |  |  |  |
| **ct 20.0% tail (start)** | 5.06E-112 | 1.42E-250 | 9.68E-84 | 1.53E-65 | 1.26E-115 | 2.16E-27 | 2.11E-175 | **0.00E+00** | 5.57E-122 |
| **ct 20.0% tail (end)** | 3.08E-62 | 5.32E-99 | 9.48E-03 | 8.74E-19 | 1.06E-67 | 1.61E-13 | 1.01E-71 | 7.96E-164 | 9.81E-14 |
| **25 20.0% tail (start)** | 4.79E-112 | 9.18E-50 | 4.56E-181 | 1.54E-65 | 5.19E-14 | 1.50E-83 | 2.06E-175 | 2.47E-61 | 2.76E-269 |
| **25 20.0% tail (end)** | 9.75E-35 | 2.44E-04 | 2.78E-45 | 5.18E-21 | **2.89E-01** | 1.46E-52 | 4.54E-54 | 6.84E-03 | 6.33E-95 |
| **75 20.0% tail (start)** | 5.79E-15 | 2.35E-228 | 3.67E-149 | **3.28E-01** | 1.77E-100 | 3.14E-87 | 5.73E-10 | **0.00E+00** | 8.43E-235 |
| **75 20.0% tail (end)** | 2.22E-08 | 5.28E-49 | 3.81E-23 | 8.14E-08 | 3.12E-22 | 1.74E-03 | 2.14E-14 | 6.72E-56 | 4.36E-15 |
