# Supplementary material for: The Progeny of Arabidopsis thaliana Plants Exposed to Salt Exhibit Changes in DNA Methylation, Histone Modifications and Gene Expression
Source: PLoS One. 2012 Jan 23;7(1):e30515. doi: 10.1371/journal.pone.0030515 (PMC3264603; doi:10.1371/journal.pone.0030515)
Supplement: Table S3 — Sequence polymorphism between C24 and Columbia genomes. TAIR database was used for the analysis of sequence polymorphism. Table shows the gene ID, gene symbol, number of polymorphisms in each gene, type of substitutions, gene length and percentage of polymorphism. (DOCX) [file pone.0030515.s008.docx]

**Table S3. Sequence polymorphism between C24 and Columbia genomes**

| **Gene ID** | **Gene Symbol** | **Number of mutations** | **Substitution type** | **Gene length** | **% of polymorphism** |
| --- | --- | --- | --- | --- | --- |
| AT2G23740 | SUVH6 | 3 | (G🡪A, T🡪G, C🡪G) | 5761 | 0.052 |
| AT2G24740 | SUVH8 | 3 | (T🡪C, G🡪A, C🡪T) | 2268 | 0.132 |
| AT2G33290 | SUVH2 | 0 |  | 2424 | 0 |
| AT2G35160 | SUVH5 | 4 | (G🡪A, G🡪T, C🡪G, G🡪A) | 3339 | 0.120 |
| AT4G02150 | MOS6 | 0 |  | 3080 | 0 |
| AT3G48900 | UVH3-homologue | 0 |  | 4515 | 0 |
| AT2G29140 | APUM3 | 1 | (C🡪T) | 4562 | 0.022 |
| AT2G28380 | DRB2 | 2 | (A🡪C, G🡪T) | 2370 | 0.084 |
| **Average** |  | **1.63** |  | **3540** | **0.051** |
