# Supplementary material for: The Progeny of Arabidopsis thaliana Plants Exposed to Salt Exhibit Changes in DNA Methylation, Histone Modifications and Gene Expression
Source: PLoS One. 2012 Jan 23;7(1):e30515. doi: 10.1371/journal.pone.0030515 (PMC3264603; doi:10.1371/journal.pone.0030515)
Supplement: Table S4 — Primers used for the analysis of histone modifications and gene expression. “ChiP primers” – primers used for amplification of immunoprecipitated DNA. ‘qPCR” – primers used for the analysis of gene expression. “SUVH6-p-for” and “SUVH6-p-rev” – forward and reverse primers used for amplification of promoter regions. “SUVH6-t-for” and “SUVH6-t-rev” – forward and reverse primers used for amplification of the transcript (coding) regions. (DOCX) [file pone.0030515.s009.docx]

**Table S4. Primers used for the analysis of histone modifications and gene expression**

|  | **ChIP primers** | **qPCR primers** |
| --- | --- | --- |
| SUVH6-p-for | TTATCCCTATTCCCTAGCATA |  |
| SUVH6-p-rev | GTGTGATTCTTATCTTCTTCTG |  |
| SUVH6-t-for | TTCGCCACAAGGATTATC | |
| SUVH6-t-rev | CATTCTCTGGTGTATTCATTAC | |
| SUVH8-p-for | ATTGCTTGACTAATGTTTCA |  |
| SUVH8-p-rev | CAGAATAGACTTATCGGTTG |  |
| SUVH8-t-for |  | ACATCAGCACCTCCTCAT |
| SUVH8-t-rev |  | CCAGCACTCGCATCATAA |
| DRB2-p-for | GCTTACAATAGTGGTGGATTATAG |  |
| DRB2-p-rev | CGCTGCTAGTCAACTGAA |  |
| DRB2-t-for | ACCAACCTGTGTTTACTG | |
| DRB2-t-rev | CTTCTCTGCTTGCTTCTT | |
| APUM3-p-for | CCCAGTTTCTTCTTAAAGTTTC |  |
| APUM3-p-rev | AAATCCTAAAGATGACACCTT |  |
| APUM3-t-for | ATAGTGGAAGTGGAGTAG | |
| APUM3-t-rev | TCATACATTGGAGAATAGTTAT | |
| SUVH2-p-for | ACCAAATAATTAGTACAGAAGAA |  |
| SUVH2-p-rev | GTATGAACTTAAGATCGGAAT |  |
| SUVH2-t-for | TTATTCGTATCTCAGAGC | |
| SUVH2-t-rev | CAGAATCCAATCCGTATA | |
| SUVH5-p-for | GAAGCCGAACGGGTGATA |  |
| SUVH5-p-rev | TGATTCAATGTTGCATGATCTAGG |  |
| SUVH5-t-for | ACGACATTACAATCATCAG | |
| SUVH5-t-rev | CTTGAAGACGAGTTTACC | |
| ROS1-p-for | AGAAGAAACGAAGCATCA |  |
| ROS1-p-rev | CAGTAGAATCAATGGTTATGG |  |
| ROS1-t-for |  | ACCTGCTTCTCTAATGTC |
| ROS1-t-rev |  | AACTTCAACTCGTCCTAA |
| UVH3 homologue-p-for | TGCTATGTGCCTGGTAAT |  |
| UVH3 homologue-p-rev | AATTCTTCACTTCGGTTCG |  |
| UVH3 homologue-t-for | TTCGTGCTATATTGGTTC | |
| UVH3 homologue-t-for | AATAACTTTCGCCTCTTT | |
| UBP26-p-rev | CGAGTTTATTGGGACATT |  |
| UBP26-p-rev | CGCTCTCTTATTTCAGATT |  |
| UBP26-t-for | TGTTAGAGGCATCTGACT | |
| UBP26-t-for | CAGGTTTCCATAATTTGTTCT | |
| WRKY22-p-for | GTAATGAAGCAGAACCAA |  |
| WRKY22-p-rev | AATAATCCGTCAGCAGTA |  |
| WRKY22-t-for | CGACCACTATTGCTACTTAT | |
| WRKY22-t-rev | GCTAGATGATCCTCAACAG | |
| MSH6-p-for | GAGAGCGAGTATTATTAC |  |
| MSH6-p-rev | ATTATGGAGTGAAGAGAT |  |
| MSH6-t-for | GGTAATGTGGAAGAAGATA | |
| MSH6-t-rev | ATTCTCATCAACCAACTC | |
| MOS6-p-for | GAGTGGCAGGTTCGTTAT |  |
| MOS6-p-for | CAAGAGCGTGTACTTAGGA |  |
| MOS6-t-for |  | GAAATGTTGCTGGAGACT |
| MOS6-t-for |  | TTGAATTGAGACAGAAGAGG |
| Actin7-for | CAGTCCAAGAGAGGTATC |  |
| Actin7-rev | AAGTGTGATGCCATATCT |  |
| RCE1-for |  | CTGATGCATGGATATTACC |
| RCE1-rev |  | ACTGTGTTAATGTTAAAGAA |
| Tubulin-for |  | CTCAAGAGGTTCTCAGCAGTA |
| Tubulin-rev |  | TCACCTTCTTCATCCGCAGTT |
