# Supplementary material for: The Progeny of Arabidopsis thaliana Plants Exposed to Salt Exhibit Changes in DNA Methylation, Histone Modifications and Gene Expression
Source: PLoS One. 2012 Jan 23;7(1):e30515. doi: 10.1371/journal.pone.0030515 (PMC3264603; doi:10.1371/journal.pone.0030515)
Supplement: Table S5 — Correlation analysis performed between the following parameters: “prom-H3K9Ac”, “prom-H3K9me2”, “gene-H3K9Ac”, “gene-H3K9me2”, ‘mRNA”. (DOCX) [file pone.0030515.s010.docx]

**Table S5. Correlation analysis**

|  |  |  | |  |  |  |
| --- | --- | --- | --- | --- | --- | --- |
| **SUVH6** | prom-H3K9Ac | prom-H3K9me2 | | gene-H3K9Ac | gene-H3K9me2 | mRNA |
| prom-H3K9Ac |  | 0.52 | | 0.97 | -0.64 | -0.30 |
| prom-H3K9me2 | |  | | 0.29 | 0.32 | -0.97 |
| gene-H3K9Ac |  |  | |  | -0.81 | -0.05 |
| gene-H3K9me2 |  |  | |  |  | -0.54 |
| Methylation | -0.79 | 0.11 | | -0.92 | 0.98 | -0.35 |
| **SUVH8** | prom-H3K9Ac | prom-H3K9me2 | | gene-H3K9Ac | gene-H3K9me2 | mRNA |
| prom-H3K9Ac |  | 0.24 | |  |  | -0.87 |
| prom-H3K9me2 | |  | |  |  | -0.69 |
| gene-H3K9Ac |  |  | |  |  |  |
| gene-H3K9me2 |  |  | |  |  |  |
| Methylation | 1.00 | 0.24 | |  |  | -0.87 |
| **SUVH2** | prom-H3K9Ac | prom-H3K9me2 | | gene-H3K9Ac | gene-H3K9me2 | mRNA |
| prom-H3K9Ac |  | -0.93 | | 0.61 | -0.85 | 0.91 |
| prom-H3K9me2 | |  | | -0.28 | 0.99 | -1.00 |
| gene-H3K9Ac |  |  | |  | -0.11 | 0.23 |
| gene-H3K9me2 |  |  | |  |  | -0.99 |
| Methylation | -0.09 | 0.45 | | 0.73 | 0.59 | -0.49 |
| **SUVH5** | prom-H3K9Ac | prom-H3K9me2 | | gene-H3K9Ac | gene-H3K9me2 | mRNA |
| prom-H3K9Ac |  | -0.08 | | 0.99 | 0.17 | 0.99 |
| prom-H3K9me2 | |  | | -0.18 | -1.00 | -0.22 |
| gene-H3K9Ac |  |  | |  | 0.26 | 1.00 |
| gene-H3K9me2 |  |  | |  |  | 0.30 |
| Methylation | 0.02 | 0.99 | | -0.08 | -0.98 | -0.11 |
| **ROS1** | prom-H3K9Ac | prom-H3K9me2 | | gene-H3K9Ac | gene-H3K9me2 | mRNA |
| prom-H3K9Ac |  | -0.61 | |  |  | 0.68 |
| prom-H3K9me2 | |  | |  |  | -1.00 |
| gene-H3K9Ac |  |  | |  |  |  |
| gene-H3K9me2 |  |  | |  |  |  |
| Methylation | -0.95 | 0.33 | |  |  | -0.42 |
| **UBP26** | prom-H3K9Ac | prom-H3K9me2 | | gene-H3K9Ac | gene-H3K9me2 | mRNA |
| prom-H3K9Ac |  | -0.18 | | 0.37 | -0.38 | 0.74 |
| prom-H3K9me2 | |  | | -0.38 | 0.98 | -0.80 |
| gene-H3K9Ac |  |  | |  | -1.00 | 0.90 |
| gene-H3K9me2 |  |  | |  |  | -0.91 |
| Methylation | -0.02 | 0.99 | | -0.94 | 0.93 | -0.69 |
| **WRKY22** | prom-H3K9Ac | prom-H3K9me2 | | gene-H3K9Ac | gene-H3K9me2 | mRNA |
| prom-H3K9Ac |  | -0.70 | | 0.90 | 0.01 | -0.83 |
| prom-H3K9me2 | |  | | 0.01 | 0.70 | 0.19 |
| gene-H3K9Ac |  |  | |  | 0.44 | -0.99 |
| gene-H3K9me2 |  |  | |  |  | -0.56 |
| Methylation | 0.00 | 0.72 | | 0.43 | 1.00 | -0.55 |
| **MSH6** | prom-H3K9Ac | prom-H3K9me2 | | gene-H3K9Ac | gene-H3K9me2 | mRNA |
| prom-H3K9Ac |  | -1.00 | | 0.94 | -0.94 | 0.83 |
| prom-H3K9me2 | |  | | -0.94 | 0.94 | -0.82 |
| gene-H3K9Ac |  |  | |  | -1.00 | 0.97 |
| gene-H3K9me2 |  |  | |  |  | -0.97 |
| Methylation | -0.89 | | 0.88 | -0.99 | 0.99 | -0.99 |
| **UVH3-like** | prom-H3K9Ac | prom-H3K9me2 | | gene-H3K9Ac | gene-H3K9me2 | mRNA |
| prom-H3K9Ac |  | -0.96 | | 0.92 | -0.09 | 0.99 |
| prom-H3K9me2 | |  | | -0.09 | -0.20 | -0.99 |
| gene-H3K9Ac |  |  | |  | 0.30 | 0.97 |
| gene-H3K9me2 |  |  | |  |  | 0.04 |
| Methylation | -0.53 | 0.75 | | -0.81 | -0.80 | -0.64 |
| **DRB2** | prom-H3K9Ac | prom-H3K9me2 | | gene-H3K9Ac | gene-H3K9me2 | mRNA |
| prom-H3K9Ac |  |  | | 0.84 |  | 0.28 |
| prom-H3K9me2 | |  | |  |  |  |
| gene-H3K9Ac |  |  | |  |  | 0.76 |
| gene-H3K9me2 |  |  | |  |  |  |
| Methylation | -0.78 |  | | -1.00 |  | -0.82 |
| **APUM3** | prom-H3K9Ac | prom-H3K9me2 | | gene-H3K9Ac | gene-H3K9me2 | mRNA |
| prom-H3K9Ac |  |  | | 0.68 |  | 0.12 |
| prom-H3K9me2 | |  | |  |  |  |
| gene-H3K9Ac |  |  | |  |  | 0.81 |
| gene-H3K9me2 |  |  | |  |  |  |
| Methylation | -0.94 |  | | -0.89 |  | -0.45 |
| **MOS6** | prom-H3K9Ac | prom-H3K9me2 | | gene-H3K9Ac | gene-H3K9me2 | mRNA |
| prom-H3K9Ac |  |  | |  |  | 0.53 |
| prom-H3K9me2 | |  | |  |  |  |
| gene-H3K9Ac |  |  | |  |  |  |
| gene-H3K9me2 |  |  | |  |  |  |
| Methylation | -0.94 |  | |  |  | -0.79 |
