# Supplementary material for: The Progeny of Arabidopsis thaliana Plants Exposed to Salt Exhibit Changes in DNA Methylation, Histone Modifications and Gene Expression
Source: PLoS One. 2012 Jan 23;7(1):e30515. doi: 10.1371/journal.pone.0030515 (PMC3264603; doi:10.1371/journal.pone.0030515)
Supplement: Table S6 — Comparison of methylation, gene expression and histone modifications data. “Number” – the gene number; “Promoter/gene” – the region analyzed; “Symbol” – a gene symbol; “methylation” – the region in which methylation difference between the progeny of stressed and control plants was identified (“Promoter 75 hyper” – means that the progeny of plants exposed to 75 mM NaCl were hypermethylated at the promoter region); “expression” – the mRNA level in the progeny of plants exposed to either 25 mM or 75 mM NaCl as compared to the progeny of control plants, “−”– the lower level, “+”– the higher level, “ = ”– a similar level of expression; “H3K9ac” and “H3K9me2” – the level of specific modification in the promoter and gene body regions in the progeny of stressed plants compared to the progeny of control plants. (DOCX) [file pone.0030515.s011.docx]

| **Number** |  | **Promoter/gene** | **Symbol** | **Methylation** | | **Expression** | | **H3K9ac** | | **H3K9me2** | |
| --- | --- | --- | --- | --- | --- | --- | --- | --- | --- | --- | --- |
|  | |  |  |  | | 25 mM | 75 mM | 25 mM | 75 mM | 25 mM | 75 mM |
| at2g24740 | | Promoter | SUVH8 | Promoter 75_hyper | | - | - | = | + | + | + |
| at2g33290 | | Gene | SUVH2 | Promoter 75_hyper | | - | - | - | = | + | + |
|  | | Promoter |  |  |  |  |  | - | - | + | + |
| at2g35160 | | Gene | SUVH5 | Promoter 75_hyper | | - | - | - | - | + | + |
|  | | Promoter |  |  |  |  |  | - | - | = | - |
| at2g23740 | | Gene | SUVH6 | Promoter 75_hyper | | = | - | - | - | + | + |
|  | | Promoter |  |  |  |  |  | - | - | = | + |
| at4g01250 | | Gene | WRKY22 | Promoter 75_hyper | | + | = | - | = | = | + |
|  | | Promoter |  |  |  |  |  | - | - | + | + |
| at4g02070 | | Gene | MSH6 | Promoter 25_hyper | | - | - | - | - | + | + |
|  | | Promoter |  |  |  |  |  | - | - | + | + |
| at2g36490 | | Promoter | ROS1 | Promoter 25_hyper | | - | - | - | - | + | + |
| at3g48900 | | Gene | UVH3 homologue | Promoter 25_hypo | | + | = | + | + | + | + |
|  | | Promoter |  |  |  |  |  | + | = | - | - |
| at4g02150 | | Promoter | MOS6 (importin alpha) | Promoter 25_hyper | | - | - | - | - |  |  |
| at3g49600 | | Gene | UBP26 | Gene 25_hyper | | - | = | - | - | + | + |
|  | | Promoter |  |  |  |  |  | - | + | + | + |
| at2g28380 | | Gene | DRB2 | Gene 25_hyper | | = | - | - | - |  |  |
|  | | Promoter |  |  |  |  |  | - | - |  |  |
| at2g29140 | | Gene | APUM3 | Gene 25_hyper | | - | - | - | - |  |  |
|  | | Promoter |  |  |  |  |  | - | - |  |  |

**Table S6. Comparison of methylation, gene expression and histone modifications data**
